# Flower preferences and pollen transport networks for cavity‐nesting solitary bees: Implications for the design of agri‐environment schemes

**DOI:** 10.1002/ece3.4234

**Published:** 2018-07-07

**Authors:** Catherine E. A. Gresty, Elizabeth Clare, Dion S. Devey, Robyn S. Cowan, Laszlo Csiba, Panagiota Malakasi, Owen T. Lewis, Katherine J. Willis

**Affiliations:** ^1^ Department of Zoology University of Oxford Oxford UK; ^2^ School of Biological and Chemical Sciences Queen Mary University of London London UK; ^3^ Royal Botanic Gardens, Kew Richmond UK

**Keywords:** agri‐environment schemes, pollen transport networks, solitary bees, wildflower seed mixtures

## Abstract

Floral foraging resources are valuable for pollinator conservation on farmland, and their provision is encouraged by agri‐environment schemes in many countries. Across Europe, wildflower seed mixtures are widely sown on farmland to encourage pollinators, but the extent to which key pollinator groups such as solitary bees exploit and benefit from these resources is unclear. We used high‐throughput sequencing of 164 pollen samples extracted from the brood cells of six common cavity‐nesting solitary bee species (*Osmia bicornis*,* Osmia caerulescens*,* Megachile versicolor*,* Megachile ligniseca*,* Megachile centuncularis* and *Hylaeus confusus*) which are widely distributed across the UK and Europe. We documented their pollen use across 19 farms in southern England, UK, revealing their forage plants and examining the structure of their pollen transport networks. Of the 32 plant species included currently in sown wildflower mixes, 15 were recorded as present within close foraging range of the bees on the study farms, but only *Ranunculus acris* L. was identified within the pollen samples. *Rosa canina* L. was the most commonly found of the 23 plant species identified in the pollen samples, suggesting that, in addition to providing a nesting resource for *Megachile* leafcutter bees, it may be an important forage plant for these species. Higher levels of connectance and nestedness were characteristic of pollen transport networks on farms with abundant floral resources, which may increase resilience to species loss. Our data suggest that plant species promoted currently by agri‐environment schemes are not optimal for solitary bee foraging. If a diverse community of pollinators is to be supported on UK and European farmland, additional species such as *R. canina* should be encouraged to meet the foraging requirements of solitary bees.

## 1 INTRODUCTION

The conservation of pollinators is an issue of global concern, influencing agricultural policy at all levels from smallholdings to industrial scale farming (Byrne & Fitzpatrick, 2009). The European Food Safety Authority has estimated that pollinators contribute hundreds of millions of dollars to the European Union (EU) agricultural industry and are responsible for 80% of wild and commercial pollination services across the EU. Yet pollinators are at risk from multiple stressors, and there are significant knowledge gaps concerning the effectiveness of conservation practices targeted at pollinator communities (EFSA 2014). Within the United Kingdom, the National Pollinator Strategy is a 10‐year plan to support pollinating insects which strongly emphasizes enhancing the availability of floral foraging resources across urban and rural landscapes (DEFRA, 2014). It is vital that bees and other pollinating insects have access to floral foraging resources throughout the adult flight season to meet their energetic requirements and maximize reproductive output (Müller et al., 2006; Vaudo, Tooker, Grozinger, & Patch, 2015). However, wildflower foraging resources have declined across much of the British countryside over the last century, a trend which is thought to have been a major contributor to long‐term declines in bee populations (Baude et al., 2016; Brown & Paxton, 2009; Goulson, Lye, & Darvill, 2008; Vanbergen, 2013).

A major driver of the reduction in wildflower foraging resources has been the conversion of natural and semi‐natural flower‐rich habitat to intensive farmland. Across the UK, approximately 97% of flower‐rich grasslands were lost over the course of the 20th century (Howard, Watkins, Clarke, Barnett, & Stark, 2003). To mitigate the impact of agricultural intensification on floral resource availability, UK agri‐environment schemes now emphasize the provision of floral foraging resources for bees and other insect pollinators (DEFRA, 2014; Natural England, 2016). Two strategies are used to achieve this (Dicks et al., 2015): the conservation of flower‐rich habitats such as species‐rich grasslands and hedgerows, and the sowing of wildflower seed mixtures containing species believed to be attractive to insect pollinators (Natural England, 2016; Supporting Information Table S1).

While these interventions support the foraging requirements of bumblebees and honeybees (Carvell, Meek, Pywell, Goulson, & Nowakowski, 2007; Edwards, 2003; Holland, Smith, Storkey, Lutman, & Aebischer, 2015; Pywell et al., 2005; Rundlöf, Nilsson, & Smith, 2008), the impact on other important insect pollinators is unclear (Wood, Holland, & Goulson, 2015). For example, the plant species included in some mixes have been selected predominantly on the basis of honeybee and bumblebee foraging data (Haaland, Naisbit, & Bersier, 2011), but may be of limited utility to other wild insect pollinators (Wood et al., 2015) such as the 225 species of British solitary bees (Dicks, Showler, & Sutherland, 2010; Dicks et al., 2015; Garibaldi et al., 2014; Wood et al., 2015).

Solitary bees are the most species‐rich group of bees within the UK; of the 250 bee species present in Britain, 225 species are solitary (Falk, 2015). Solitary bees are divided into ground and cavity‐nesting species. Ground‐nesting species, such as *Adrena* spp. create subterranean nesting chambers by burrowing into the ground, often selecting south‐facing patches of sparsely vegetated, sandy soil (Falk, 2015). By contrast, cavity nesters such as *Osmia* spp. typically nest aboveground, using hollow plant stems and cavities in dead wood or mortar in walls (Michener, 2000). Cavity‐nesting species will also readily occupy artificial solitary bee nesting boxes. Most solitary bees are polylectic, obtaining pollen from several plant families; examples include *Osmia bicornis* L. and *Megachile centuncularis* L. A smaller number of solitary bee species are oligolectic, using a narrower range of plants; an example is *Andrena humilis* Imhoff, 1832, which forages on yellow Asteraceae such as Cat's ear (*Hypochaeris radicata* L.). A few species are monolectic and forage on a single plant species; for example, *Macropis europaea* Warncke, 1973, which forages exclusively on Yellow Loosestrife, *Lysimachia vulgaris* L. (Falk, 2015). Polylectic species are much more likely to benefit from sown wildflower resources than oligolectic and monolectic species, unless their specific forage plants are represented. There is a strong association between dietary specialization and vulnerability to population decline among solitary bees: oligolectic and monolectic species have declined significantly in Britain over recent decades, to a much greater extent than more generalist polylectic species (Biesmeijer et al., 2006).

The pollination service provided by solitary bees is substantial. In a recent review, the value of the global pollination service provided by wild bees was estimated at $3,251/ha, similar to the contribution of managed honeybees, and seven of the ten wild bee species identified as providing the highest mean contribution to crop production were solitary (Kleijn et al., 2015). For some important pollinator‐dependent crops grown in the UK, such as apple, solitary bees are more efficient pollinators than honeybees (Delaplane, Mayer, & Mayer, 2000; Garratt et al., 2014). As the cultivation of crops that depend on insect pollination continues to expand (Aizen, Garibaldi, Cunningham, & Klein, 2008), the conservation of solitary bee communities on farmland is important; it is estimated that current honeybee populations are capable of supplying just 34% of the pollination service demand in the UK (Breeze, Bailey, Balcombe, & Potts, 2011). The effectiveness of current wildflower mixes in supporting solitary bee foraging in the UK landscape remains a key knowledge gap and one that this study aimed to address, for cavity‐nesting solitary bees specifically.

To identify the foraging preferences of pollinators, and to construct plant‐pollinator interaction networks, most studies have used observational approaches based on the frequency of flower visitation (Carreck & Williams, 2002; Winfree, Williams, Gaines, Ascher, & Kremen, 2008; Wood et al., 2015). As observational approaches typically yield a small sample of interaction data over a limited geographic area and timescale, such studies are sometimes combined with analysis of pollen loads from insect samples, using light microscopy to identify the pollen carried. However, it is often challenging to identify pollen to species level using light microscopy, particularly for families such as Asteraceae and Brassicaceae, which are widely used by bees (Williams & Kremen, 2007). In addition, the process is extremely time consuming and requires a high level of experience and expertise in morphological pollen identification.

An alternative approach is to sequence pollen DNA from the bodies of pollinators, or from the brood cells of nesting bees (e.g., Bell et al., 2017; Lucas et al., 2018; Pompanon et al., 2012). While plant identification from DNA has presented numerous challenges (CBOL 2009), it has the advantage of providing finer definition of species‐specific foraging preferences for plant families whose pollen is difficult to distinguish morphologically (Bell et al., 2016; Bruni et al., 2015; Hawkins et al., 2015; Kraaijeveld et al., 2015; Pornon, Andalo, Burrus, & Escaravage, 2017). DNA sequencing has been used successfully to identify the foraging preference of honeybees and the floral composition of their honey (Bruni et al., 2015; De Vere et al., 2017; Galimberti et al., 2014; Hawkins et al., 2015; Jain, Jesus, Marchioro, & Araújo, 2013; Valentini, Miquel, & Taberlet, 2010). Several studies have also used pollen DNA sequencing to identify solitary bee foraging preferences (Sickel et al., 2015; Wilson, Sidhu, Levan, & Holway, 2010). One challenge where DNA may provide a solution is to resolve interactions when the diversity of plants visited is high. For example, two recent studies comparing honeybee pollen use on the basis of light microscopy versus DNA sequencing found that, although the methods agreed on the main plant families and genera involved, DNA sequencing provided higher species resolution (Keller et al., 2015; Smart et al., 2017). Another recent study comparing pollen metabarcoding with observation of flower visits found that metabarcoding revealed 2.5 times more plant species (Pornon et al., 2016). While some of these species may have been detected from very small quantities of pollen or DNA including contact contamination in nature and therefore not represent preferred plant species; overall, the increase in resolution is extremely valuable in providing more detailed insight into foraging patterns (Pornon et al., 2017). In a small number of cases, DNA based approaches have been incorporated into interaction networks and have demonstrated that this new level of resolution can provide dramatic changes in network metrics (Wirta et al., 2014). While DNA has been used to resolve seed dispersal networks (e.g., García‐Robledo, Erickson, Staines, Erwin, & Kress, 2013; González‐Varo, Arroyo, & Jordano, 2014), to our knowledge this approach has not previously been applied to pollen transport networks.

In this paper, we use pollen metabarcoding to evaluate the suitability of forage resource provision in UK agri‐environmental schemes, focusing on plant use and plant‐pollinator interaction networks for cavity‐nesting solitary bees.

Our objectives were to (a) identify the key plant species that cavity‐nesting solitary bees visit and use to provision their offspring; and (b) quantify pollinator‐pollen interaction networks for cavity‐nesting solitary bees on farmland managed under a gradient of investment in agri‐environment management. Our data allow us to assess, for the subset of common cavity‐nesting solitary bees that we sampled, whether the sown wildflower seed mixtures of the agri‐environment schemes support their foraging preferences.

In addition, our data set allows us to examine whether agri‐environment management, through the provision of floral foraging resources, influences the structure of the pollen transport networks of these bee species. At a community level, the structure and dynamics of ecological networks are thought to be a key determinant of stability and closely related to the delivery of ecosystem functions and services (McCann, 2000; Montoya, Pimm, & Solé, 2006; Thébault & Fontaine, 2010). We were interested to examine, for these interaction networks, whether key network metrics related to stability were influenced positively by agri‐environment management.

## 2 MATERIALS AND METHODS

### 2.1 Study sites

Nineteen farms were selected within a 60‐mile radius of Oxford (UK), ranging in size from 33 to 1,626 ha (see Supporting Information Table S2). The farms were chosen to span a gradient of investment in agri‐environment interventions, ranging from no interventions to active management targeted at enhancing pollinator foraging resource availability. These included providing flower‐rich field margins, actively sowing wildflower seed mixtures containing species presumed to be attractive to pollinators and maintaining species‐rich grassland. Interventions were associated with Nature England's categorization of “Entry Level Stewardship” and “Organic Entry Level Stewardship” (the more basic agri‐environment agreements) and “Higher Level Stewardship” and “Organic Higher Level Stewardship” (more comprehensive agreements) (DEFRA). For a list of all interventions to enhance foraging resource availability for insect pollinators under these agri‐environment agreements, see Supporting Information Table S1. In summary, farms managed under Entry Level Stewardship will typically invest in fewer of these interventions over a smaller geographic area while farms under Higher Level Stewardship will typically have greater investment. Sixteen farms were mixed arable and pastoral, and three were entirely pastoral.

### 2.2 Solitary bee nest boxes

Fifteen solitary bee nest boxes were placed on each of the 19 farms in March 2014, so that they were available in time for colonization by early‐emerging cavity‐nesting solitary bees and wasps (Falk, 2015). Nest boxes followed designs used successfully in previous studies (Fabian et al., 2013; Steckel et al., 2014; Tylianakis, Tscharntke, & Lewis, 2007; Williams & Kremen, 2007). Each consisted of a 20 cm long plastic cylinder, of diameter 10 cm, filled with hollow, untreated sections of bamboo with apertures ranging from 4 to 12 mm in diameter (Falk, 2015; Tylianakis et al., 2007). Each nest was attached perpendicular to a 1.5 m vertical wooden stake, with the entrance to the nest box oriented to the south. The 15 trap nests were placed approximately equidistant across the whole of each farm (at least 20 m apart in all directions), the local criterion being that the nest had to be situated on a south‐facing field margin as bees prefer nesting sites exposed to direct sunlight (Falk, 2015). The nest boxes were, as a consequence of this design, further apart on the larger farms; however, in the original selection of farms, we were careful to ensure a range of farm sizes within each stewardship category to ensure that farm size and management regime were not confounded. The nest boxes were collected in November following the end of solitary bee flight season (Falk, 2015).

### 2.3 Pollinator foraging resource availability

The abundance and species richness of floral foraging resources were recorded on each farm in spring (April–May 2014), early‐midsummer (June–July 2014) and late summer‐early autumn (August–September 2014). All flowering entomophilous plants within a 15 m radius of each nest box were identified to species. The abundance of floral units of each species was recorded on a scale of 1–5, using the DAFOR Scale where 1 is rare and 5 is dominant (Hill, 2005). Floral units were categorized as a single solitary flower or capitulum (e.g., *Leucanthemum vulgare* Lam.), cyme (e.g., *Myosotis arvensis* (L.) Hill.), raceme (e.g., *L. vulgaris* L.), umbel (e.g., *Anthriscus sylvestris* (L.) Hoffm.), corymb (e.g., *Jacobea vulgaris* P. Gaertn.*)* or panicle (e.g., *Centranthus ruber* (L.) DC.) as appropriate, following Clapham, Tutin, and Moore (1990), Rose, O'Reilly, Smith, and Collings (2006) and Stace (2010) (see Supporting Information Table S7). Data for each farm were pooled to assess the overall floral‐unit abundance. As nectar and pollen provided by floral units varies across plant species, values are an approximate estimate of foraging resource availability.

### 2.4 Collection of pollen samples

In November 2014, all occupied bamboo tubes were extracted from the solitary bee nest boxes. Each tube was opened to record the total number of brood cells present and to collect samples of residual brood cell pollen. For each tube, it was assumed that a single female bee was responsible for all of the brood cells. Pollen samples were collected and pooled from the brood cells at either end of the tube (i.e., the oldest and youngest brood cells) to represent pollen collected at the beginning and end of the female's flight season.

### 2.5 Rearing and identification of bees

The bamboo tubes were re‐sealed after pollen collection and placed over winter in a climatically controlled room at 5°C. In March 2015, they were transferred to room temperature to stimulate adult emergence. Bees emerging from the bamboo tubes were identified to species and released. Only pollen samples from bamboo tubes from which at least one bee successfully emerged were selected for pollen analysis, resulting in 164 analyzed pollen samples.

### 2.6 Next generation sequencing of pollen sample DNA

The pollen samples were cleaned in 70% ethanol and DNA was extracted using the DNeasy Plant Mini Kit (Qiagen), following the manufacturer's protocol.

PCR amplification of the internal transcribed spacer region 2 (ITS2) was carried out on the individual samples using the ITS3 and ITS4 primers (White, Bruns, Lee, & Taylor, 1990). The PCR reaction mix contained 25 μl of Q5^®^ High‐Fidelity 2× Master Mix (New England Biolabs), 2.5 μl each of the ITS3 and ITS4 primer, 2 μl of genomic DNA and 18 μl of nuclease‐free water. The cycling program was 98°C 30 s, {98°C 10 s, 63°C 30 s, 72°C 30 s} for 32 cycles, 72°C 2 min. The products were cleaned using the UltraClean 96 PCR Cleanup Kit (384) (Qiagen) following the manufacturer's protocol and measured using a Quantus™ Fluorometer (Promega).

Amplicon libraries were prepared using 200 ng of each PCR product and the NEBNext^®^ Ultra™ II DNA Library Prep Kit for Illumina^®^ with dual indexing (barcoding). The products were end repaired and “A” tailed using the NEBNext End Prep module, NEBNext adaptors were ligated onto the amplicons using the NEB Adaptor Ligation Module and dual indexes incorporated during the PCR enrichment step using NEBNext Ultra ll Q5 Master Mix and NEB Next Multiplex Oligos for Illumina. The final products were cleaned using Ampure XP beads and pooled into 2 batches of 80 samples.

The pooled samples were normalized to 4 nM, denatured with 0.2 M NaOH, diluted with loading buffer to 17.5 pM and run on as a 2 × 300 paired‐end read on a MiSeq using Reagent Kit v3 (600‐cycles). PhiEx was added to the pooled samples (5%) to provide the required variability for adequate cluster recognition on the MiSeq flowcell.

A reference collection was generated using samples from the DNA bank at Kew or samples collected from species recorded on the study farms. These were Sanger sequenced bidirectionally using the same initial PCR primers and parameters as for the libraries. This reference database was converted to a BLAST database using makeblastdb in the BLAST suit (Bethesda, 2008).

Processing of the FastQ output files included assembling reads in MOTHUR (Kozich, Westcott, Baxter, Highlander, & Schloss, 2013) and adapter removal using CUTADAPT (Martin, 2011) with any reads that could not be assembled or with incomplete adaptors excluded from further analysis. The reads were further screened to remove those with ambiguous base calls or longer than 389 bp (suggesting incorrect assembly). Because the region is variable in length, it was necessary to determine a suitable length parameter for screening the assembly (advocated by the MOTHUR pipeline). We determined these values following an iterative process of testing different lengths, starting with small values similar to those suggested by MOTHUR's WIKI SOP (250 bp). The maxambig and maxlength parameters and summary.seqs command were used to increase sequence length gradually, retaining as much data as possible without retaining sequences with a large number of ambiguous base calls or runs of identical bases (polymers), which suggest poor quality (Kozich et al., 2013); this may, however, exclude some rare taxa with very long sequences in this region. The reads were reduced to unique haplotypes for identification.

The data were compared to the reference database using a local BLAST method and the results were parsed using custom python scripts, which retain only the top 10 matches for each read. Fungal matches and other obvious contaminants were removed. Only matches which showed 100% similarity to the reference database were considered. This represents a conservative approach to taxonomic assignment and will remove low‐quality matches and putative chimeric sequences. One possible issue with the BLAST approach is that a sequence may generate multiple perfect matches if taxa in the reference database are indistinguishable. To minimize this we used a high bit score (bit score >600) which eliminated most matches that had low query coverage and we examined the reference database using both sequence alignments and neighbor‐joining trees to identify taxa which cannot be reliably separated using this region (e.g., species with the same haplotype). For example, species within the genus *Veronica* are indistinguishable in our reference dataset, so matches to those references are retained only at genus level. Following all processing steps, 1,580 haplotypes were retained representing 136 of the original 164 samples; the rest produced no matches meeting these criteria. This process should minimize ambiguous outcomes where a haplotype is identified simultaneously as two taxa (i.e., two perfect 100% matches for one haplotype retained).

### 2.7 Network visualization

Quantitative pollen transport networks were constructed in R 2.14.0 (R Core Team, 2013) using the bipartite package (Dormann, Fründ, Blüthgen, & Gruber, 2009) for the 15 farms for which pollen samples, identifiable to specific bee species, were available. As this high‐throughput sequence method cannot quantify the abundance of species in each sample the pollen transport networks are quantified instead based on the number of pollen samples in which each plant species was identified (i.e., incidence), for each bee species.

### 2.8 Network metrics

Quantitative (weighted) network metrics were calculated for each farm following the methods outlined by (Bersier, Banašek‐Richter, & Cattin, 2002), using the bipartite package (Dormann et al., 2009) in R 2.14.0 (R Core Team, 2013). The following weighted network metrics were selected as they have been identified as good indicators of stability for pollination networks (Bascompte, Jordano, Melián, & Olesen, 2003; Bastolla et al., 2009; Tylianakis, Laliberté, Nielsen, & Bascompte, 2010):

1. *Connectance* **:** The fraction of all possible links within a network that are realized. The higher the level of network connectance, the greater the degree of generalism of the plant and pollinator species involved. This is thought to confer greater resilience to species loss, as there is more flexibility for individuals to switch interaction partner, limiting the risk of a cascade of secondary species extinctions (Dunne, Williams, & Martinez, 2002; Montoya et al., 2006; Thébault & Fontaine, 2010). A quantitative, weighted measure of connectance (Bersier et al., 2002) was selected to take into account the relative frequency of interactions between plants and pollinators.
2. *Linkage Density:* The mean number of links per species within the network. Higher link density is believed to correlate with greater resilience to species loss in a similar way to network connectance (Dunne et al., 2002; Montoya et al., 2006; Thébault & Fontaine, 2010). A weighted measure of link density was selected to take into account the relative frequency of interactions (Bersier et al., 2002).
3. *Generality (bee species):* The mean number of plant species visited by each pollinator species within the network, again weighted to take into account the relative frequency of interactions (Bersier et al., 2002). Higher generality is thought to increase network stability by decreasing the vulnerability of pollinator species to perturbations in the population size of the plant species that they visit. If one or more interactions are disrupted, the impact will be lower for more generalist pollinators that are able to visit many plant species, conferring greater resilience to plant species loss (McCann, 2000; Tylianakis et al., 2010).
4. *Nestedness:* The extent to which the more specialist pollinator species within the network interact with subsets of the plant species with which more generalist pollinators interact, weighted to take into account the relative frequency of interactions (Bastolla et al., 2009). Higher levels of nestedness are believed to enhance community stability by ensuring a redundancy of interactions: If one pollinator species goes extinct, the plant species that it visited will still be pollinated by other species (Bascompte et al., 2003; Bastolla et al., 2009).

Generalized linear models were constructed in R 2.14.0 (R Core Team, 2013) to investigate the effect of the explanatory variables floral‐unit abundance, plant species richness, number of pollen samples available and number of bee species from which pollen samples were taken on the value of each network response metric. Metrics were calculated for the 14 study farms where interactions were documented between a minimum of two plant and two bee species. The number of pollen samples available for each farm and the number of bee species from which pollen samples were taken were included as explanatory variables to ensure differences in sample size across farms were taken into account.

The ggpubr R package was used to visualize the relationships between these variables to confirm that the covariation was linear. Gaussian error structures were specified for all models. Models including all explanatory variables, but no interactions, were constructed in the first instance and then simplified following the method outlined by Crawley (2005), using the Akaike Information Criterion (AIC), to identify minimum adequate models. Values of AIC were calculated in R 2.14.0 using the MASS package (Venables & Ripley, 2013). AIC values were also calculated for the intercept only models in each case in order to ascertain that the AIC value of the best‐fit models were >2 points smaller than the intercept only models. Examination of the model validation plots indicated that, in each case, the model assumptions were met.

## 3 RESULTS

### 3.1 Nest box occupancy

In total, 255 solitary bee nest boxes were recovered from the farms, containing a total of 275 occupied bamboo tubes and 2,204 solitary bee brood cells. At least one bee emerged successfully from 164 of the occupied bamboo tubes, with six species recorded: *O. bicornis* (Linnaeus, 1758), *Osmia caerulescens* (Linnaeus, 1758), *Megachile versicolor* Smith, F., 1844., *Megachile ligniseca* (Kirby, 1802), *M. centuncularis* (Linnaeus, 1758), and *Hylaeus confusus* Nylander, 1852.

### 3.2 Floral foraging resource availability

The number of flowering potential foraging plant species ranged from 27 to 67 species per farm. There was a significant positive correlation between farm agri‐environment management level and both the abundance of floral units (Spearman's Correlation, *r*_s_(17) = 0.546, *p*‐value = 0.017) and their species richness (Spearman's Correlation, *r*_s_(17) = 0.603, *p*‐value = 0.006). A Spearman's correlation was selected in place of a linear model because of the small number of farms within each agri‐environment level category (there were only four farms in the no Stewardship and Entry Level Stewardship categories). The farms were ranked according to the level of Environmental Stewardship agreement, from farms under no stewardship scheme to those showcasing Higher Level agreements, with organically managed farms ranked above the conventional ones within each level of stewardship category. If there was more than organic or conventional farm in each category, the farms were ranked according to the proportion of natural and semi‐natural habitat present.

Plant species richness correlated significantly with floral‐unit abundance across all farms (Pearson product‐moment correlation, *r*(17) = 0.803, *p*‐value <0.001). Visualization of the relationship between the two variables as a scatter plot, using the ggpubr package in R, confirmed that the covariation was linear and Shapiro–Wilk normality tests confirmed both variables followed a normal distribution; demonstrating the assumptions of the Pearson correlation were met. In total, 15 of the 34 plant species included in the sown wildflower seed mixes of the agri‐environment schemes were recorded on the 15 study farms from which brood cell pollen samples were sequenced successfully. Overall, these 15 plant species constituted 11.9% of total floral‐unit abundance (Table 1).

**Table 1:** Plant species included currently in the sown wildflower seed mixtures of the UK agri‐environment schemes (Carvell, Meek, Pywell, & Nowakowski, 2004; Carvell et al., 2007; Pywell et al., 2011; Wood et al., 2017) and their representation across study farms

Species included currently in sown wildflower seed mixtures	Recorded on study farms	% Of total floral‐unit abundance (across all study farms)
*Achillea millefolium* L.	No	–
*Centaurea cyanus* L.	Yes	0.08
*Centaurea nigra* L.	Yes	0.73
*Centaurea scabiosa* L.	No	–
*Daucus carota* L.	No	–
*Galium verum* L.	Yes	0.09
*Geranium pratense* L.	Yes	0.13
*Knautia arvensis* L.	Yes	0.26
*Lathyrus pratensis* L.	No	–
*Leontodon hispidus* L.	No	–
*Leucanthemum vulgare* Lam.	Yes	0.91
*Lotus corniculatus* L.	Yes	1.13
*Lotus pedunculatus* Cav.	No	–
*Lychnis flos‐cuculi* L.	Yes	0.05
*Malva moschata* L.	No	–
*Medicago lupulina* L.	Yes	1.55
*Medicago sativa* L.	No	–
*Melilotus officinalis* L.	No	–
*Onobrychis viciifolia* L.	Yes	0.10
*Origanum vulgare* L.	Yes	0.03
*Phacelia tanacetifolia* Benth.	No	–
*Plantago lanceolate* L.	No	–
*Plantago media* L.	No	–
*Primula veris* L.	Yes	0.48
*Prunella vulgaris* L.	Yes	0.11
*Ranunculus acris* L.	Yes	5.01
*Rhinanthus minor* L.	No	–
*Rumex acetosella* L.	No	–
*Sanguisorba minor* L.	No	–
*Silene dioica* L.	Yes	0.36
*Sonchus arvensis* L	Yes	1.96
*Trifolium hybridum* L.	No	–

### 3.3 Pollen species identification and transport networks

**Figure 1:**
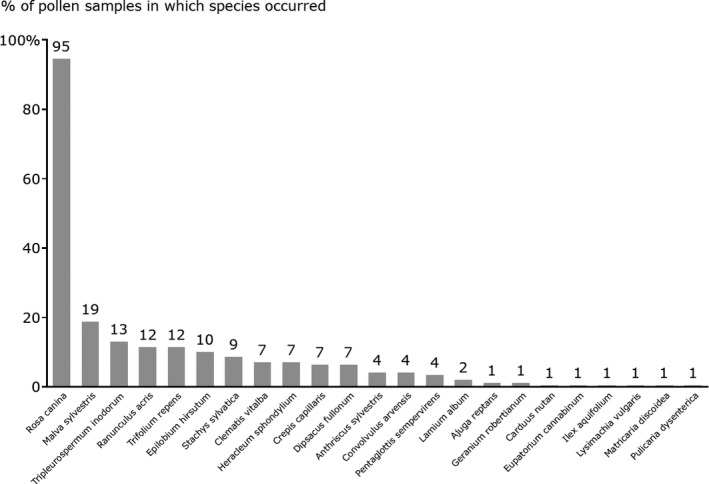
Bar chart displaying, for all 136 pollen samples sequenced successfully, the percentage of samples, in which each plant species was recorded

**Figure 2:**
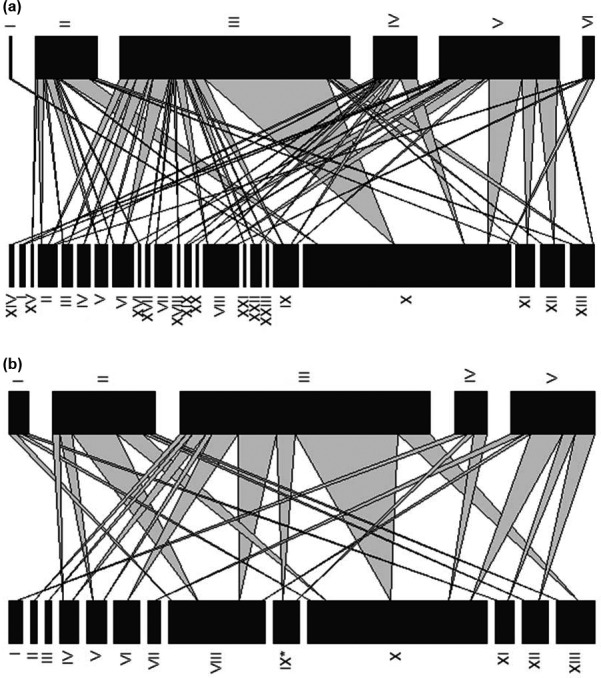
Pollen transport network for all farms. The lower bars represent the plant species and the upper bars represent bee species. Linkage width indicates the fraction of pollen samples from that bee species, in which the plant species occurred. The length of the bars for the bee species represents the number of pollen samples sequenced for each species. The length of the bars for plant species represents the frequency of occurrence of each species within the pollen samples. For bee species I *Hylaeus confusus,* II *Megachile ligniseca,* III *Megachile versicolor,* IV *Osmia bicornis,* V *Osmia caerulescens, &* VI *Megachile centuncularis*. *For plant species* I *Anthriscus sylvestris,* II *Clematis vitalba,* III *Convolvulus arvensis,* IV *Crepis capillaris,* V *Dipsacus fullonum,* VI *Epilobium hirsutum,* VII *Heracleum sphondylium,* VIII *Malva sylvestris,* IX *Ranunculus acris,* X *Rosa canina,* XI *Stachys sylvatica,* XII *Trifolium repens,* XIII *Tripleurospermum inodorum,* XIV *Ajuga reptans,* XV *Carduus nutans,* XVI *Eupatorium cannabinum,* XVII *Geranium robertianum,* XVIII *Ilex aquifolium,* XIX *Lamium album,* XX *Lysimachia vulgaris,* XXI *Matricaria discoidea,* XXII *Pentaglottis sempervirens, and* XXIII *Pulicaria dysenterica*

Overall, 23 plant species were identified within the pollen samples (see Table 2 and Figure 1). All of these had been recorded as present on the study farms, but only one species currently sown within wildflower seed mixtures (*Ranunculus acris* L.)*,* was identified within the pollen samples. Pollen transport networks were constructed for each farm (see Supporting Information Figure S2a–n) and the networks were pooled for the no Stewardship and Entry Level Stewardship farms (see Figure 2a), for comparison with the Higher Level Stewardship Farms (Figure 2b) to allow comparison of network structures between farms under low and high agri‐environment scheme management levels. See Supporting Information Table S6, for the full pollen DNA output data, listing the pollen sample IDs (corresponding to the nest box and bamboo shoot the pollen was extracted from), the farm the pollen sample was taken from, the bee species the pollen sample came from and the plant species identified from the DNA analysis.

**Table 2:** Plant species identified within the pollen samples

	*Osmia bicornis*	*Osmia caerulescens*	*Megachile versicolor*	*Megachile ligniseca*	*Megachile centuncularis*	*Hylaeus confusus*
No. pollen samples analysed	12	23	78	18	3	2
*Rosa canina* L.	83.30	91.30	97.44	100.00	100.00	50.00
*Malva sylvestris* L.	16.70	43.48	12.82	5.60	66.66	50.00
*Tripleurospermum inodorum* (L.) Sch.Bip	0.00	4.35	16.67	22.20	0.00	0.00
*Ranunculus acris* L.*	33.30	8.70	10.26	5.60	0.00	50.00
*Trifolium repens* L.	0.00	56.52	2.56	5.60	0.00	0.00
*Epilobium hirsutum* L.	0.00	4.35	15.38	5.60	0.00	0.00
*Stachys sylvatica* L.	0.00	39.13	1.28	5.60	0.00	50.00
*Clematis vitalba* L.	8.30	0.00	6.41	16.70	33.33	0.00
*Heracleum sphondylium* L.	0.00	39.13	1.28	0.00	0.00	0.00
*Dipsacus fullonum* L.	0.00	0.00	3.85	33.30	0.00	0.00
*Crepis capillaris* (L.) Wallr	0.00	0.00	10.26	5.60	0.00	0.00
*Anthriscus sylvestris* (L.) Hoffm.	33.30	8.70	0.00	0.00	0.00	0.00
*Convolvulus arvensis* L.	0.00	0.00	6.41	5.60	0.00	0.00
*Pentaglottis sempervirens* (L.) Tausch ex L.H.Bailey	16.70	0.00	3.85	0.00	0.00	0.00
*Lamium album* L.	8.30	8.70	0.00	0.00	0.00	0.00
*Ajuga reptans* L.	0.00	8.70	0.00	0.00	0.00	0.00
*Geranium robertianum* L.	8.30	0.00	1.28	0.00	0.00	0.00
*Carduus nutans* L.	0.00	0.00	0.00	5.60	0.00	0.00
*Eupatorium cannabinum* L.	0.00	0.00	0.00	5.60	0.00	0.00
*Ilex aquifolium* L.	0.00	0.00	1.28	0.00	0.00	0.00
*Lysimachia vulgaris* L.	8.30	0.00	0.00	0.00	0.00	0.00
*Matricaria discoidea* DC.	0.00	0.00	1.28	0.00	0.00	0.00
*Pulicaria dysenterica* (L.) Bernh	0.00	0.00	1.28	0.00	0.00	0.00

*Note*

For each bee species, the percentage of pollen samples sequenced for that species in which each plant occurred is displayed. The cells are shaded according to this % value. *Ranunculus acris* is marked with an asterisk as this is the only plant species from the list included currently in the agri‐environment scheme wildflower seed mixtures. The plant species are ranked according to the number of pollen samples each was present in, from highest to lowest.

The weighted network metrics connectance, linkage density, generality (bees) and nestedness were calculated for the 14 farms, where sufficient data were available (see Supporting Information Table S3). None of the network metrics differed significantly between farms with low versus higher levels of agri‐environment agreement (no stewardship and Entry Level Stewardship farms vs. Higher Level Stewardship farms) (One‐way ANOVAs, see Supporting Information Table S4 and Figure S1).

### 3.4 Across‐farm analyses of network metrics

#### 3.4.1 Connectance

The best‐fitting model included floral‐unit abundance, plant species richness and the number of bee species as explanatory variables. Connectance increased significantly with floral‐unit abundance (*F*_1,12_ = 2.497, *p*‐value <0.007), plant species richness (*F*_1,11_ = 23.394, *p*‐value = 0.004) and the number of bee species from which pollen samples were taken (GLM, *F*_1,10_ = 51.167, *p*‐value <0.001).

#### 3.4.2 Linkage density

The best‐fitting model included the number of bee species from which pollen samples were taken as the sole explanatory variable. Although the number of bee species was not significantly associated with linkage density (GLM, *F*_1,12_ = 3.369, *p*‐value = 0.0913), the AIC value for this best‐fit model was <2 points from the AIC value for the intercept only model; therefore we assume, conservatively, that this model had limited explanatory power.

#### 3.4.3 Generality (bees)

The best‐fitting model included plant species richness as the sole explanatory variable. Plant species richness had no significant effect on Generality (bees) (GLM, *F*_1,12_ = 2.754, *p*‐value = 0.123). Again, however, the AIC value for this best‐fit model was within <2 points of the AIC value for the intercept only model; thus we assume that this model also had limited explanatory power.

#### 3.4.4 Nestedness

The best‐fitting model included floral‐unit abundance as the sole explanatory variable. Nestedness increased significantly with floral‐unit abundance (GLM, *F*_1,10_ = 29.953, *p*‐value <0.001).

#### 3.4.5 Number of plant species visited within farm networks

The best‐fitting model included the number of bee species from which pollen samples were taken, floral‐unit abundance and plant species richness as explanatory variables. The number of bee species had a positive effect on the number of plant species visited within the networks (GLM, *F*_1,11_ = 9.356, *p*‐value = 0.011). Floral‐unit abundance had no significant effect (GLM, *F*_1,13_ = 2.685, *p*‐value = 0.223) and neither did plant species richness (GLM, *F*_1,12_ = 5.098, *p*‐value = 0.175).

For further detail on model output and selection, including AIC scores, for all of these GLMs, see Supporting Information Table S5.

## 4 DISCUSSION

Our data document the effect of farm management on the foraging networks of six species of solitary bee: *O. bicornis*,* O. caerulescens*,* M. versicolor*,* M. ligniseca*,* M. centuncularis* and *H. confusus*. All of these species are widely distributed across much of the UK and commonly occupy solitary bee nest boxes; *O. bicornis* is also an important pollinator of oil seed rape and apples (Falk, 2015; Gruber, Eckel, Everaars, & Dormann, 2011). These bee species have wide distributions extending across Europe and beyond (BWARS, 2018), so the foraging data that we present here has the potential to inform conservation measures more widely within European agri‐environment schemes. Our data suggest that the current formulation of wildflower seed mixtures to support pollinators is not optimal for these bee species, and alternative plants such as *R. canina* should be encouraged to support their foraging requirements more specifically.

### 4.1 Foraging preferences

While some data are available on the foraging plants used by these bee species in the UK (Falk, 2015), our data set allows more detailed examination of their foraging behavior within agricultural landscapes specifically, and their use of plants used within sown wildflower seed mixtures, of relevance to the design of effective farmland conservation interventions. Although fifteen of the plant species included currently in the UK agri‐environment scheme sown wildflower mixes were recorded on the study farms, only one of these, *R. acris* L.*,* was detected within the brood cell pollen samples of the cavity‐nesting bee species studied. This is concerning and supports recent research suggesting that many of the plant species encouraged by agri‐environment schemes are of limited utility to solitary bees (Wood, Holland, & Goulson, 2017). The plant species that occurred most frequently in pollen samples was the wild rose *R. canina* L., which was identified in 129 of the 136 samples. *Rosa canina* was not particularly abundant across the study farms, constituting just 0.12% of total floral‐unit abundance. Its frequent occurrence in pollen samples cannot therefore be explained by a high abundance in the environment.

There is evidence to suggest that solitary bees forage closer to nesting areas than do other bees (Carman & Jenkins, 2016) and therefore it is surprising to find a high abundance of *Rosa* across all sampling areas. Leafcutter bees of the genus *Megachile* often construct brood cells using rose leaves (Falk, 2015), leading to the possibility that the pollen samples of *M. versicolor*,* M. ligniseca,* and *M. centuncularis* were contaminated by brood cell leaf DNA. However, *R. canina* pollen was also identified in 91% (21 of 23) of the pollen samples from *O. caerulescens*, 83% (10 of 12) of the samples from *O. bicornis* and 50% (1 of 2) samples from *H. confusus*, species that do not use leaves in this way. DNA based methods can be extremely sensitive to very low‐level presence of a taxa and thus some natural background contamination cannot be excluded as an explanation. One of the main advantages of DNA based methodology is the sensitivity of the technique to very small quantities of DNA; however, this also makes very low‐level contamination in natural samples hard to exclude. We have attempted to use very conservative criteria in the identifications; however, the presence–absence nature of metabarcoding analyses (Pompanon et al., 2012) can also overemphasize the importance of rare or trace material. Despite these cautions, this study provides tentative evidence that *R. canina* might be a previously unknown forage plant for these bee species. Its dual use for forage and brood cell construction warrants further examination of its importance as a forage plant for cavity‐nesting solitary bees. If further study confirms these findings, there would be strong justification for its inclusion within the set of plants encouraged by agri‐environment schemes. *Rosa canina* is a climbing deciduous shrub and so is not suitable for inclusion in wildflower seed mixtures; however, it could be encouraged as a hedgerow plant. Its range extends across Europe, northwest Africa, and western Asia so, if demonstrated to provide an important forage resource for these bee species across Europe more widely, it could also be encouraged by the agri‐environment schemes of European counties.

Other frequent plant species within the pollen samples included *Malva sylvestris* L.*, Tripleurospermum inodorum* (L.) Sch.Bip*, R. acris* and *Trifolium repens* L. Again, these plant species were not present at especially high abundance on the study farms, comprising 0.05%, 0.19%*,* 5.01% and 6.01% of total floral‐unit abundance, respectively. *Malva* is a popular nectar source of bees which likely explains its high incidence as pollen could be easily acquired during feeding. However, *T. inodorum* and *T. repens* were also identified as important forage plants for solitary bees in a recent study (Wood et al., 2017) which used field observation and pollen load analysis to examine the foraging preferences of 72 species of solitary bees (including both cavity‐ and ground‐nesting species) on farms managed under the same set of agri‐environment scheme agreements in Hampshire and West Sussex, UK. *Tripleurospermum inodorum* and *T. repens* thrive on a variety of soil types and across a broad range of habitats in the UK. *Tripleurospermum inodorum* is a weed of cereal crops, but *T. repens* is a good candidate species to be encouraged under agri‐environment agreements, and (like *R. canina*) is distributed widely across Europe making it relevant to European agri‐environment schemes.

### 4.2 Interaction networks

It is important to note that the pollen transport networks constructed in this study are based on a small subset of six bee species; data from a wider sample of species must be obtained to enable a comprehensive analysis of the impact of agri‐environment management on solitary bee network properties. However, the results of this study do present preliminary evidence that the provision of a higher abundance of foraging resources within the farmed environment, through agri‐environment schemes, may have a positive influence on the stability of the pollen transport networks of these bees.

Floral‐unit abundance, plant species richness and the number of bee species from which pollen samples were obtained were identified as positively associated with network connectance. Connectance describes the fraction of all possible links within a network that are realized, and is a standard measure of food web complexity (Rooney & McCann, 2012; Tylianakis et al., 2010). The higher the level of connectance for a given network size, the greater the degree of generalism of the species involved. This is believed to confer greater resilience to species loss, as there is more flexibility for individuals to switch interaction partner, limiting the risk of a cascade of secondary species extinctions (Dunne et al., 2002; Montoya et al., 2006; Thébault & Fontaine, 2010). Farm level floral‐unit abundance and species richness were positively associated with increased connectance, suggesting that the provision of a high abundance and species richness of forage plants within the farmed environment may increase the stability of communities of cavity‐nesting bee species.

The abundance of floral units in the environment was also identified as having a significant, positive effect on the level of nestedness within the bee pollen transport networks. The degree of nestedness within a network describes the extent to which the plant species interacting with specialist pollinators are a proper subset of the plants interacting with generalists (Bastolla et al., 2009; Tylianakis et al., 2010). A nested structure is believed to enhance the stability of pollination networks by ensuring that, if a specialist pollinator goes extinct, more generalist pollinators still pollinate the plant species that the specialist visited (Memmott, Waser, & Price, 2004; Thébault & Fontaine, 2010). The significant positive relationship identified between network nestedness and the abundance of floral units again provides tentative evidence that the network stability of these bee species might be enhanced by a high local abundance of foraging resources.

## 5 CONCLUSION

The pollination networks for UK farms constructed using DNA metabarcoding provide evidence that the sown wildflower seed mixtures of the UK agri‐environment schemes do not appear to be supporting the foraging requirements of common cavity‐nesting solitary bee species as effectively as they might (Wood et al., 2015, 2017). In particular, our data suggest that *R. canina*, in addition to providing an important nesting resource for *Megachile* leafcutter bees, may be a particularly important forage plant for these bee species, and could be encouraged as a hedgerow plant within UK and European agri‐environment schemes.

Analysis of pollen transport network structure found that connectance and nestedness were influenced positively by the abundance of floral units in the environment. This suggests that the provision of floral foraging resources may enhance the stability of the communities of these bee species. If forage resource provision was to support the specific foraging requirements of these bee species more effectively, by including more plant species of preference, a stronger positive effect of floral‐unit abundance on pollen transport network stability might be observed, with positive implications for delivery of pollination services on farmland.

## CONFLICT OF INTEREST

None declared.

## AUTHOR CONTRIBUTIONS

Catherine Gresty (Department of Zoology, University of Oxford): Collected the data, carried out all analyses, and led the writing of the manuscript; Elizabeth Clare (School of Biological and Chemical Sciences, Queen Mary University of London): Analyzed and interpreted genetic data and contributed to the writing of the manuscript; Robyn Cowan (Royal Botanic Gardens, Kew): Performed genetic analysis; Laszlo Csiba (Royal Botanic Gardens, Kew): Performed genetic analysis; Dion Devey (Royal Botanic Gardens, Kew): Performed genetic analysis; Panagiota Malakasi (Royal Botanic Gardens, Kew): Performed genetic analysis; Owen Lewis (Department of Zoology, University of Oxford): Contributed to research design, fieldwork, insect rearing, and writing of the manuscript; Katherine Willis (Department of Zoology, University of Oxford): Contributed to research design and writing of the manuscript.

## DATA ACCESSIBILITY STATEMENT

Should this paper be accepted, the data will be archived as supporting information on the Molecular Ecology website and in the Dryad Digital Repository: https://doi.org/10.5061/dryad.6vb720f. This will include an excel file, containing the raw and summarized dataset and R scripts and original sequence data and interpretation files.

## References

[ece34234-bib-0001] Aizen, M. A. , Garibaldi, L. A. , Cunningham, S. A. , & Klein, A. M. (2008). Long‐term global trends in crop yield and production reveal no current pollination shortage but increasing pollinator dependency. Current Biology, 18(20), 1572–1575. 10.1016/j.cub.2008.08.066 18926704

[ece34234-bib-0002] Barbosa, A. M. , Brown, J. A. , & Jimenez‐Valvarde, A. (2016). Real R. modEvA: Model evaluation and analysis. R package version 1.3. 2. https://cran.r-project.org/web/packages/modEvA/index.html

[ece34234-bib-0003] Bascompte, J. , Jordano, P. , Melián, C. J. , & Olesen, J. M. (2003). The nested assembly of plant–animal mutualistic networks. Proceedings of the National Academy of Sciences, 100(16), 9383–9387. 10.1073/pnas.1633576100 PMC170927 12881488

[ece34234-bib-0004] Bastolla, U. , Fortuna, M. A. , Pascual‐Garcia, A. , Ferrera, A. , Luque, B. , & Bascompte, J. (2009). The architecture of mutualistic networks minimizes competition and increases biodiversity. Nature, 458(7241), 1018 10.1038/nature07950 19396144

[ece34234-bib-0005] Baude, M. , Kunin, W. E. , Boatman, N. D. , Conyers, S. , Davies, N. , Gillespie, M. A. , … Memmott, J. (2016). Historical nectar assessment reveals the fall and rise of floral resources in Britain. Nature, 530(7588), 85 10.1038/nature16532 26842058 PMC4756436

[ece34234-bib-0006] Bell, K. L. , de Vere, N. , Keller, A. , Richardson, R. T. , Gous, A. , Burgess, K. S. , & Brosi, B. J. (2016). Pollen DNA barcoding: Current applications and future prospects. Genome, 59(9), 629–640. 10.1139/gen-2015-0200 27322652

[ece34234-bib-0007] Bell, K. L. , Fowler, J. , Burgess, K. S. , Dobbs, E. K. , Gruenewald, D. , Lawley, B. , … Brosi, B. J. (2017). Applying pollen DNA metabarcoding to the study of plant–pollinator interactions. Applications in Plant Sciences, 5(6), https://www.ncbi.nlm.nih.gov/pmc/articles/PMC5499302/ 10.3732/apps.1600124 PMC5499302 28690929

[ece34234-bib-0008] Bersier, L. F. , Banašek‐Richter, C. , & Cattin, M. F. (2002). Quantitative descriptors of food‐web matrices. Ecology, 83(9), 2394–2407. 10.1890/0012-9658(2002)083[2394:QDOFWM]2.0.CO;2

[ece34234-bib-0009] Bethesda (2008). BLAST^®^ Command line applications user manual [Internet]: Building a BLAST database with local sequences. Bethesda, MD: National Center for Biotechnology Information (US) Retrieved from https://www.ncbi.nlm.nih.gov/books/NBK279688/

[ece34234-bib-0100] Biesmeijer, J. C. , Roberts, S. P. , Reemer, M. , Ohlemüller, R. , Edwards, M. , Peeters, T. , … Settele, J. (2006). Parallel declines in pollinators and insect‐pollinated plants in Britain and the Netherlands. Science, 313(5785), 351–354. 16857940 10.1126/science.1127863

[ece34234-bib-0011] Breeze, T. D. , Bailey, A. P. , Balcombe, K. G. , & Potts, S. G. (2011). Pollination services in the UK: How important are honeybees? Agriculture, Ecosystems & Environment, 142(3–4), 137–143. 10.1016/j.agee.2011.03.020

[ece34234-bib-0012] Brown, M. J. , & Paxton, R. J. (2009). The conservation of bees: A global perspective. Apidologie, 40(3), 410–416. 10.1051/apido/2009019

[ece34234-bib-0013] Bruni, I. , Galimberti, A. , Caridi, L. , Scaccabarozzi, D. , De Mattia, F. , Casiraghi, M. , & Labra, M. (2015). A DNA barcoding approach to identify plant species in multiflower honey. Food Chemistry, 170, 308–315. 10.1016/j.foodchem.2014.08.060 25306350

[ece34234-bib-0014] BWARS . (2018). Retrieved from http://www.bwars.com/

[ece34234-bib-0015] Byrne, A. , & Fitzpatrick, Ú. (2009). Bee conservation policy at the global, regional and national levels. Apidologie, 40(3), 194–210. 10.1051/apido/2009017

[ece34234-bib-0016] Carman, K. , & Jenkins, D. G. (2016). Comparing diversity to flower‐bee interaction networks reveals unsuccessful foraging of native bees in disturbed habitats. Biological Conservation, 202, 110–118. 10.1016/j.biocon.2016.08.030

[ece34234-bib-0017] Carreck, N. L. , & Williams, I. H. (2002). Food for insect pollinators on farmland: Insect visits to flowers of annual seed mixtures. Journal of Insect Conservation, 6(1), 13–23. 10.1023/A:1015764925536

[ece34234-bib-0018] Carvell, C. , Meek, W. R. , Pywell, R. F. , Goulson, D. , & Nowakowski, M. (2007). Comparing the efficacy of agri‐environment schemes to enhance bumble bee abundance and diversity on arable field margins. Journal of Applied Ecology, 44(1), 29–40.

[ece34234-bib-0019] Carvell, C. , Meek, W. R. , Pywell, R. F. , & Nowakowski, M. (2004). The response of foraging bumblebees to successional change in newly created arable field margins. Biological Conservation, 118(3), 327–339. 10.1016/j.biocon.2003.09.012

[ece34234-bib-0020] Clapham, A. R. , Tutin, T. G. , & Moore, D. M. (1990). Flora of the British Isles. Cambridge: CUP Archive.

[ece34234-bib-0021] Crawley, M. J. (2005). Statistics: An introduction using R, 2005. New York: Wiley 10.1002/9781119941750

[ece34234-bib-0022] De Vere, N. , Jones, L. E. , Gilmore, T. , Moscrop, J. , Lowe, A. , Smith, D. , … Ford, C. R. (2017). Using DNA metabarcoding to investigate honey bee foraging reveals limited flower use despite high floral availability. Scientific Reports, 7, 42838 10.1038/srep42838 28205632 PMC5311969

[ece34234-bib-0023] DEFRA . (2014). The National pollinator Strategy for bees and other pollinators in England. Retrieved from https://www.gov.uk/government/uploads/attachment_data/file/409431/pb14221-national-pollinators-strategy.pdf

[ece34234-bib-0024] Delaplane, K. S. , Mayer, D. R. , & Mayer, D. F. (2000). Crop pollination by bees. New York: Cabi 10.1079/9780851994482.0000

[ece34234-bib-0026] Dicks, L. V. , Baude, M. , Roberts, S. P. , Phillips, J. , Green, M. , & Carvell, C. (2015). How much flower‐rich habitat is enough for wild pollinators? Answering a key policy question with incomplete knowledge. Ecological Entomology, 40(S1), 22–35. 10.1111/een.12226 26877581 PMC4737402

[ece34234-bib-0027] Dicks, L. V. , Showler, D. A. , & Sutherland, W. J. (2010). Bee conservation: Evidence for the effects of interventions, Vol. 1 Exeter: Pelagic Publishing.

[ece34234-bib-0028] Dormann, C. F. , Fründ, J. , Blüthgen, N. , & Gruber, B. (2009). Indices, graphs and null models: Analyzing bipartite ecological networks. The Open Ecology Journal, 2(1), 1–18.

[ece34234-bib-0029] Dunne, J. A. , Williams, R. J. , & Martinez, N. D. (2002). Network structure and biodiversity loss in food webs: Robustness increases with connectance. Ecology Letters, 5(4), 558–567. 10.1046/j.1461-0248.2002.00354.x

[ece34234-bib-0030] Edwards, M. (2003). Aculeate conservation group annual report. Midhurst, UK: Unpublished report for the UK BAP bumblebee working group.

[ece34234-bib-0031] European Food Safety Authority (EFSA ) (2014). Towards an integrated environmental risk assessment of multiple stressors on bees: Review of research projects in Europe, knowledge gaps and recommendations. EFSA Journal, 12(3), 3594 10.2903/j.efsa.2014.3594

[ece34234-bib-0032] Fabian, Y. , Sandau, N. , Bruggisser, O. T. , Aebi, A. , Kehrli, P. , Rohr, R. P. , … Bersier, L. F. (2013). The importance of landscape and spatial structure for hymenopteran‐based food webs in an agro‐ecosystem. Journal of Animal Ecology, 82(6), 1203–1214. 10.1111/1365-2656.12103 23863136

[ece34234-bib-0033] Falk, S. J. (2015). Field guide to the bees of Great Britain and Ireland. London: British Wildlife Publishing.

[ece34234-bib-0035] Galimberti, A. , De Mattia, F. , Bruni, I. , Scaccabarozzi, D. , Sandionigi, A. , Barbuto, M. , … Labra, M. (2014). A DNA barcoding approach to characterize pollen collected by honeybees. PLoS ONE, 9(10), e109363 10.1371/journal.pone.0109363 25296114 PMC4190116

[ece34234-bib-0036] García‐Robledo, C. , Erickson, D. L. , Staines, C. L. , Erwin, T. L. , & Kress, W. J. (2013). Tropical plant–herbivore networks: Reconstructing species interactions using DNA barcodes. PLoS ONE, 8(1), e52967 10.1371/journal.pone.0052967 23308128 PMC3540088

[ece34234-bib-0037] Garibaldi, L. A. , Carvalheiro, L. G. , Leonhardt, S. D. , Aizen, M. A. , Blaauw, B. R. , Isaacs, R. , … Morandin, L. (2014). From research to action: Enhancing crop yield through wild pollinators. Frontiers in Ecology and the Environment, 12(8), 439–447. 10.1890/130330

[ece34234-bib-0038] Garratt, M. P. D. , Truslove, C. L. , Coston, D. J. , Evans, R. L. , Moss, E. D. , Dodson, C. , … Potts, S. G. (2014). Pollination deficits in UK apple orchards. Journal of Pollination Ecology, 12(2), 9–14.

[ece34234-bib-0039] González‐Varo, J. P. , Arroyo, J. M. , & Jordano, P. (2014). Who dispersed the seeds? The use of DNA barcoding in frugivory and seed dispersal studies. Methods in Ecology and Evolution, 5(8), 806–814. 10.1111/2041-210X.12212

[ece34234-bib-0041] Goulson, D. , Lye, G. C. , & Darvill, B. (2008). Decline and conservation of bumble bees. Annual Review of Entomology, 53, 191–208. 10.1146/annurev.ento.53.103106.093454 17803456

[ece34234-bib-0200] Group, C. P. W. , Hollingsworth, P. M. , Forrest, L. L. , Spouge, J. L. , Hajibabaei, M. , Ratnasingham, S. , … Fazekas, A. J. (2009). A DNA barcode for land plants. Proceedings of the National Academy of Sciences, 106(31), 12794–12797. 10.1073/pnas.0905845106 PMC2722355 19666622

[ece34234-bib-0043] Gruber, B. , Eckel, K. , Everaars, J. , & Dormann, C. F. (2011). On managing the red mason bee (Osmia bicornis) in apple orchards. Apidologie, 42(5), 564 10.1007/s13592-011-0059-z

[ece34234-bib-0044] Haaland, C. , Naisbit, R. E. , & Bersier, L. F. (2011). Sown wildflower strips for insect conservation: A review. Insect Conservation and Diversity, 4(1), 60–80. 10.1111/j.1752-4598.2010.00098.x

[ece34234-bib-0045] Hawkins, J. , de Vere, N. , Griffith, A. , Ford, C. R. , Allainguillaume, J. , Hegarty, M. J. , … Adams‐Groom, B. (2015). Using DNA metabarcoding to identify the floral composition of honey: A new tool for investigating honey bee foraging preferences. PLoS ONE, 10(8), e0134735 10.1371/journal.pone.0134735 26308362 PMC4550469

[ece34234-bib-0046] Hill D. (Ed.) (2005). Handbook of biodiversity methods: Survey, evaluation and monitoring. Cambridge: Cambridge University Press.

[ece34234-bib-0047] Holland, J. M. , Smith, B. M. , Storkey, J. , Lutman, P. J. , & Aebischer, N. J. (2015). Managing habitats on English farmland for insect pollinator conservation. Biological Conservation, 182, 215–222. 10.1016/j.biocon.2014.12.009

[ece34234-bib-0048] Howard, D. C. , Watkins, J. W. , Clarke, R. T. , Barnett, C. L. , & Stark, G. J. (2003). Estimating the extent and change in broad habitats in Great Britain. Journal of Environmental Management, 67(3), 219–227. 10.1016/S0301-4797(02)00175-5 12667472

[ece34234-bib-0049] Jain, S. A. , Jesus, F. T. D. , Marchioro, G. M. , & Araújo, E. D. D. (2013). Extraction of DNA from honey and its amplification by PCR for botanical identification. Food Science and Technology (Campinas), 33(4), 753–756. 10.1590/S0101-20612013000400022

[ece34234-bib-0052] Keller, A. , Danner, N. , Grimmer, G. , Ankenbrand, M. V. D. , Ohe, K. V. D. , Ohe, W. , … Steffan‐Dewenter, I. (2015). Evaluating multiplexed next‐generation sequencing as a method in palynology for mixed pollen samples. Plant Biology, 17(2), 558–566. 10.1111/plb.12251 25270225

[ece34234-bib-0053] Kleijn, D. , Winfree, R. , Bartomeus, I. , Carvalheiro, L. G. , Henry, M. , Isaacs, R. , … Ricketts, T. H. (2015). Delivery of crop pollination services is an insufficient argument for wild pollinator conservation. Nature Communications, 6, 7414 10.1038/ncomms8414 PMC4490361 26079893

[ece34234-bib-0054] Kozich, J. J. , Westcott, S. L. , Baxter, N. T. , Highlander, S. K. , & Schloss, P. D. (2013). Development of a dual‐index sequencing strategy and curation pipeline for analyzing amplicon sequence data on the MiSeq Illumina sequencing platform. Applied and Environmental Microbiology, 79(17), 5112–5120. 10.1128/AEM.01043-13 23793624 PMC3753973

[ece34234-bib-0055] Kraaijeveld, K. , Weger, L. A. , Ventayol García, M. , Buermans, H. , Frank, J. , Hiemstra, P. S. , & Dunnen, J. T. (2015). Efficient and sensitive identification and quantification of airborne pollen using next‐generation DNA sequencing. Molecular Ecology Resources, 15(1), 8–16. 10.1111/1755-0998.12288 24893805

[ece34234-bib-0056] Lucas, A. , Bodger, O. , Brosi, B. J. , Ford, C. R. , Forman, D. W. , Greig, C. , … de Vere, N. (2018). Floral resource partitioning by individuals within generalised hoverfly pollination networks revealed by DNA metabarcoding. Scientific Reports, 8(1), 5133 10.1038/s41598-018-23103-0 29572453 PMC5865107

[ece34234-bib-0057] Martin, M. (2011). Cutadapt removes adapter sequences from high‐throughput sequencing reads. EMBnet. Journal, 17(1), 10 10.14806/ej.17.1.200

[ece34234-bib-0058] McCann, K. S. (2000). The diversity–stability debate. Nature, 405(6783), 228 10.1038/35012234 10821283

[ece34234-bib-0059] Memmott, J. , Waser, N. M. , & Price, M. V. (2004). Tolerance of pollination networks to species extinctions. Proceedings of the Royal Society of London B: Biological Sciences, 271(1557), 2605–2611. 10.1098/rspb.2004.2909 PMC1691904 15615687

[ece34234-bib-0060] Michener, C. D. (2000). The bees of the world, Vol. 1 Baltimore: JHU press.

[ece34234-bib-0061] Montoya, J. M. , Pimm, S. L. , & Solé, R. V. (2006). Ecological networks and their fragility. Nature, 442(7100), 259 10.1038/nature04927 16855581

[ece34234-bib-0062] Müller, A. , Diener, S. , Schnyder, S. , Stutz, K. , Sedivy, C. , & Dorn, S. (2006). Quantitative pollen requirements of solitary bees: Implications for bee conservation and the evolution of bee–flower relationships. Biological Conservation, 130(4), 604–615. 10.1016/j.biocon.2006.01.023

[ece34234-bib-0063] Natural England . (2016). [Online] Countryside Stewardship. Retrieved from https://www.gov.uk/government/collections/countryside-stewardship-get-paid-for-land-management#higher-tier

[ece34234-bib-0064] Pompanon, F. , Deagle, B. E. , Symondson, W. O. , Brown, D. S. , Jarman, S. N. , & Taberlet, P. (2012). Who is eating what: Diet assessment using next generation sequencing. Molecular Ecology, 21(8), 1931–1950. 10.1111/j.1365-294X.2011.05403.x 22171763

[ece34234-bib-0065] Pornon, A. , Andalo, C. , Burrus, M. , & Escaravage, N. (2017). DNA metabarcoding data unveils invisible pollination networks. Scientific Reports, 7(1), 16828 10.1038/s41598-017-16785-5 29203872 PMC5715002

[ece34234-bib-0066] Pornon, A. , Escaravage, N. , Burrus, M. , Holota, H. , Khimoun, A. , Mariette, J. , … Vidal, M. (2016). Using metabarcoding to reveal and quantify plant‐pollinator interactions. Scientific Reports, 6, 27282 10.1038/srep27282 27255732 PMC4891682

[ece34234-bib-0068] Pywell, R. F. , Meek, W. R. , Loxton, R. G. , Nowakowski, M. , Carvell, C. , & Woodcock, B. A. (2011). Ecological restoration on farmland can drive beneficial functional responses in plant and invertebrate communities. Agriculture, Ecosystems & Environment, 140(1–2), 62–67. 10.1016/j.agee.2010.11.012

[ece34234-bib-0069] Pywell, R. F. , Warman, E. A. , Carvell, C. , Sparks, T. H. , Dicks, L. V. , Bennett, D. , … Sherwood, A. (2005). Providing foraging resources for bumblebees in intensively farmed landscapes. Biological Conservation, 121(4), 479–494. 10.1016/j.biocon.2004.05.020

[ece34234-bib-0070] R Core Team . (2013). R: A language and environment for statistical computing. R Foundation for Statistical Computing: Vienna.

[ece34234-bib-0071] Rooney, N. , & McCann, K. S. (2012). Integrating food web diversity, structure and stability. Trends in Ecology & Evolution, 27(1), 40–46. 10.1016/j.tree.2011.09.001 21944861

[ece34234-bib-0072] Rose, F. , O'Reilly, C. , Smith, D. P. , & Collings, M. (2006). The wild flower key: How to identify wild flowers, trees and shrubs in Britain and Ireland. London: Frederick Warne.

[ece34234-bib-0073] Rundlöf, M. , Nilsson, H. , & Smith, H. G. (2008). Interacting effects of farming practice and landscape context on bumble bees. Biological Conservation, 141(2), 417–426. 10.1016/j.biocon.2007.10.011

[ece34234-bib-0074] Sickel, W. , Ankenbrand, M. J. , Grimmer, G. , Holzschuh, A. , Härtel, S. , Lanzen, J. , … Keller, A. (2015). Increased efficiency in identifying mixed pollen samples by meta‐barcoding with a dual‐indexing approach. BMC Ecology, 15(1), 20 10.1186/s12898-015-0051-y 26194794 PMC4509727

[ece34234-bib-0075] Smart, M. D. , Cornman, R. S. , Iwanowicz, D. D. , McDermott‐Kubeczko, M. , Pettis, J. S. , Spivak, M. S. , & Otto, C. R. V. (2017). A comparison of honey bee‐collected pollen from working agricultural lands using light microscopy and ITS metabarcoding. Environmental Entomology, 46(1), 38–49. 28062536 10.1093/ee/nvw159

[ece34234-bib-0076] Stace, C. (2010). New flora of the British Isles. Cambridge: Cambridge University Press.

[ece34234-bib-0077] Steckel, J. , Westphal, C. , Peters, M. K. , Bellach, M. , Rothenwoehrer, C. , Erasmi, S. , … Steffan‐Dewenter, I. (2014). Landscape composition and configuration differently affect trap‐nesting bees, wasps and their antagonists. Biological Conservation, 172, 56–64. 10.1016/j.biocon.2014.02.015

[ece34234-bib-0078] Thébault, E. , & Fontaine, C. (2010). Stability of ecological communities and the architecture of mutualistic and trophic networks. Science, 329(5993), 853–856. 10.1126/science.1188321 20705861

[ece34234-bib-0079] Tylianakis, J. M. , Laliberté, E. , Nielsen, A. , & Bascompte, J. (2010). Conservation of species interaction networks. Biological Conservation, 143(10), 2270–2279. 10.1016/j.biocon.2009.12.004

[ece34234-bib-0080] Tylianakis, J. M. , Tscharntke, T. , & Lewis, O. T. (2007). Habitat modification alters the structure of tropical host–parasitoid food webs. Nature, 445(7124), 202 10.1038/nature05429 17215842

[ece34234-bib-0081] Valentini, A. , Miquel, C. , & Taberlet, P. (2010). DNA barcoding for honey biodiversity. Diversity, 2(4), 610–617. 10.3390/d2040610

[ece34234-bib-0082] Vanbergen, A. J. , & Insect Pollinators Initiative (2013). Threats to an ecosystem service: Pressures on pollinators. Frontiers in Ecology and the Environment, 11(5), 251–259. 10.1890/120126

[ece34234-bib-0083] Vaudo, A. D. , Tooker, J. F. , Grozinger, C. M. , & Patch, H. M. (2015). Bee nutrition and floral resource restoration. Current Opinion in Insect Science, 10, 133–141. 10.1016/j.cois.2015.05.008 29588000

[ece34234-bib-0084] Venables, W. N. , & Ripley, B. D. (2013). Modern applied statistics with S‐PLUS. New York: Springer Science & Business Media.

[ece34234-bib-0085] White, T. J. , Bruns, T. , Lee, S. J. W. T. , & Taylor, J. L. (1990). Amplification and direct sequencing of fungal ribosomal RNA genes for phylogenetics. PCR Protocols: A Guide to Methods and Applications, 18(1), 315–322.

[ece34234-bib-0086] Williams, N. M. , & Kremen, C. (2007). Resource distributions among habitats determine solitary bee offspring production in a mosaic landscape. Ecological Applications, 17(3), 910–921. 10.1890/06-0269 17494406

[ece34234-bib-0087] Wilson, E. E. , Sidhu, C. S. , Levan, K. E. , & Holway, D. A. (2010). Pollen foraging behaviour of solitary Hawaiian bees revealed through molecular pollen analysis. Molecular Ecology, 19(21), 4823–4829. 10.1111/j.1365-294X.2010.04849.x 20958818

[ece34234-bib-0088] Winfree, R. , Williams, N. M. , Gaines, H. , Ascher, J. S. , & Kremen, C. (2008). Wild bee pollinators provide the majority of crop visitation across land‐use gradients in New Jersey and Pennsylvania. USA. Journal of Applied Ecology, 45(3), 793–802.

[ece34234-bib-0089] Wirta, H. K. , Hebert, P. D. , Kaartinen, R. , Prosser, S. W. , Várkonyi, G. , & Roslin, T. (2014). Complementary molecular information changes our perception of food web structure. Proceedings of the National Academy of Sciences, 111(5), 1885–1890. 10.1073/pnas.1316990111 PMC3918760 24449902

[ece34234-bib-0090] Wood, T. J. , Holland, J. M. , & Goulson, D. (2015). Pollinator‐friendly management does not increase the diversity of farmland bees and wasps. Biological Conservation, 187, 120–126. 10.1016/j.biocon.2015.04.022

[ece34234-bib-0091] Wood, T. J. , Holland, J. M. , & Goulson, D. (2017). Providing foraging resources for solitary bees on farmland: Current schemes for pollinators benefit a limited suite of species. Journal of Applied Ecology, 54(1), 323–333. 10.1111/1365-2664.12718

