# Supplementary material for: Flower preferences and pollen transport networks for cavity‐nesting solitary bees: Implications for the design of agri‐environment schemes
Source: Ecol Evol. 2018 Jul 7;8(15):7574–87. doi: 10.1002/ece3.4234 (PMC6106195; doi:10.1002/ece3.4234)
Supplement: Supplementary file 1 [file ECE3-8-7574-s001.docx]

**Flower preferences and pollen transport networks for cavity nesting solitary bees: implications for the design of agri-environment schemes**

**^1^ Catherine E. A. Gresty (corresponding author)**

76 Barrowgate Road, Chiswick, London W4 4QU

**^2^ Elizabeth Clare**

School of Biological and Chemical Sciences, Queen Mary University of London, Mile End Rd., London, E1 4NS, United Kingdom

**^3^ Dion S. Devey**

Royal Botanic Gardens, Kew, Richmond, Surrey TW9 3AE, United Kingdom

**^4^ Robyn S. Cowan**

Royal Botanic Gardens, Kew, Richmond, Surrey TW9 3AE, United Kingdom

**^5^ Laszlo Csiba**

Royal Botanic Gardens, Kew, Richmond, Surrey TW9 3AE, United Kingdom

**^6^ Panagiota Malakasi**

Royal Botanic Gardens, Kew, Richmond, Surrey TW9 3AE, United Kingdom

**^7^ Owen T. Lewis**

Department of Zoology, New Radcliffe House, Radcliffe Observatory Quarter, 6GG, Woodstock Rd, Oxford OX2

**^8^ Katherine J. Willis**

Department of Zoology, New Radcliffe House, Radcliffe Observatory Quarter, 6GG, Woodstock Rd, Oxford OX2; Royal Botanic Gardens, Kew, Richmond, Surrey TW9 3AE, United Kingdom

**Supplementary Information**


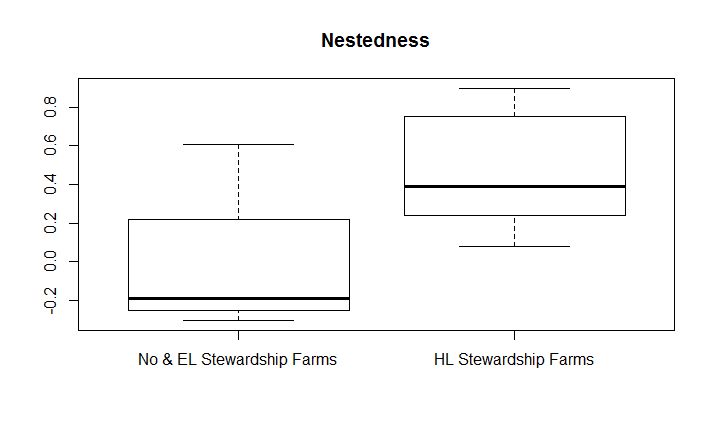

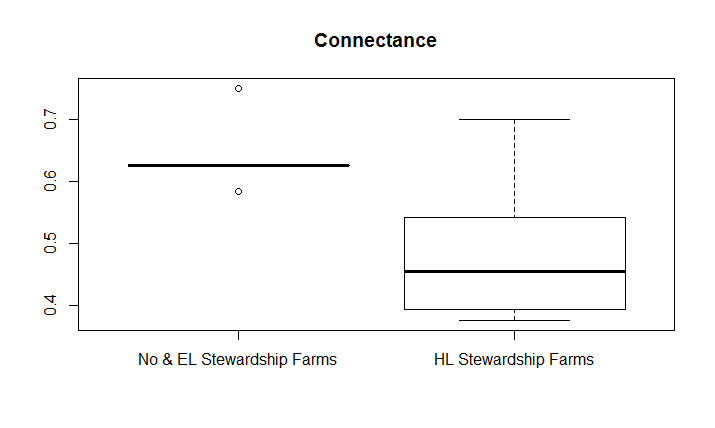


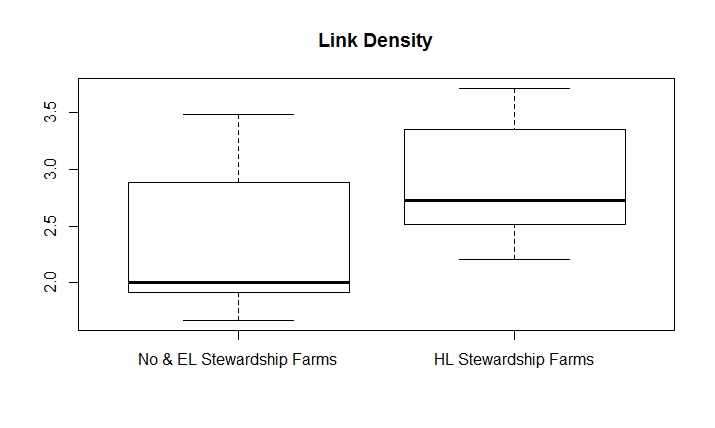

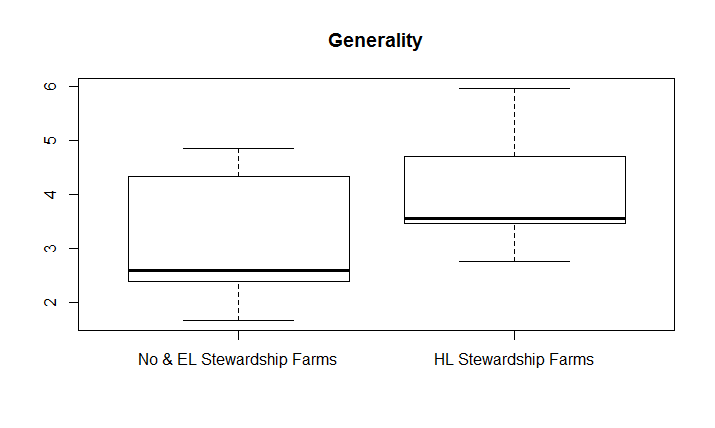


**Figure 1** Box plots visualising comparison of network metrics between farms under low agri-environment management (No Stewardship & Entry Level Stewardship farms) and high agri-environment management (Higher Level Stewardship Farms)


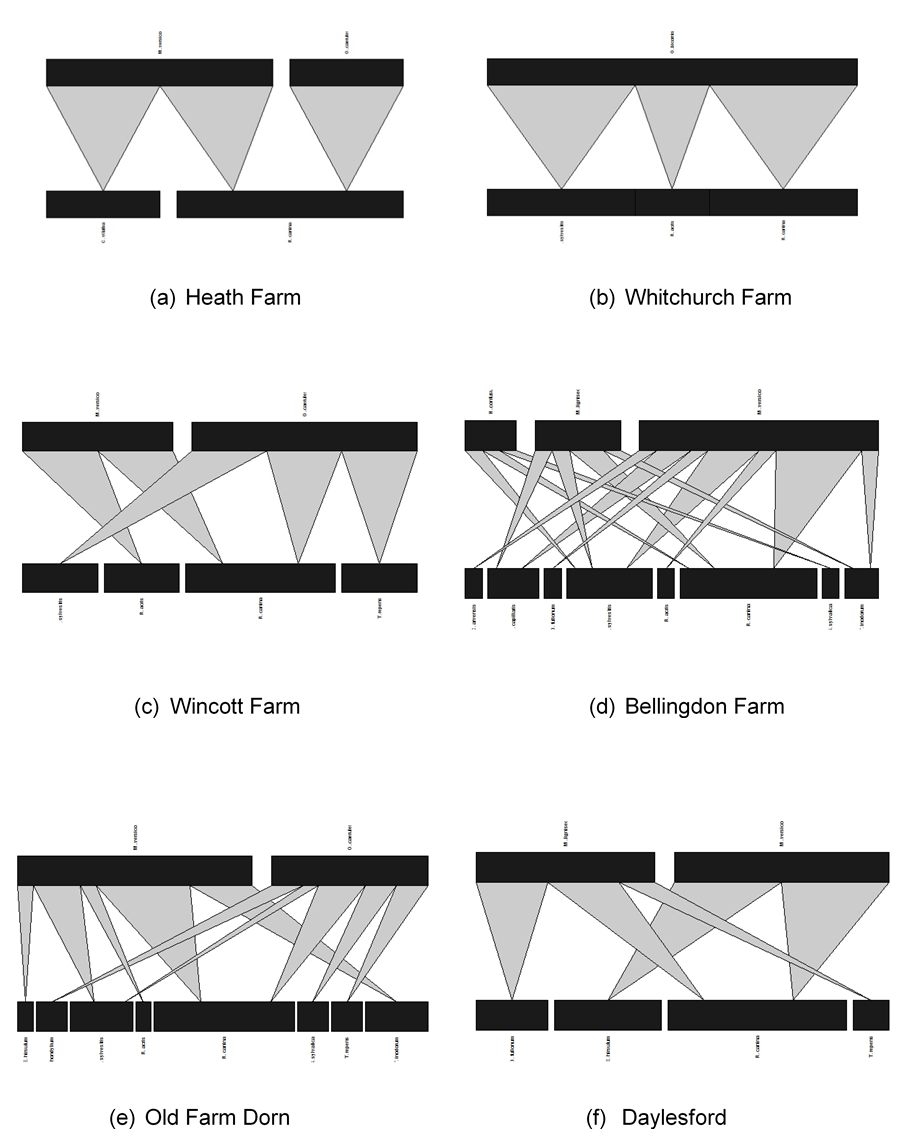


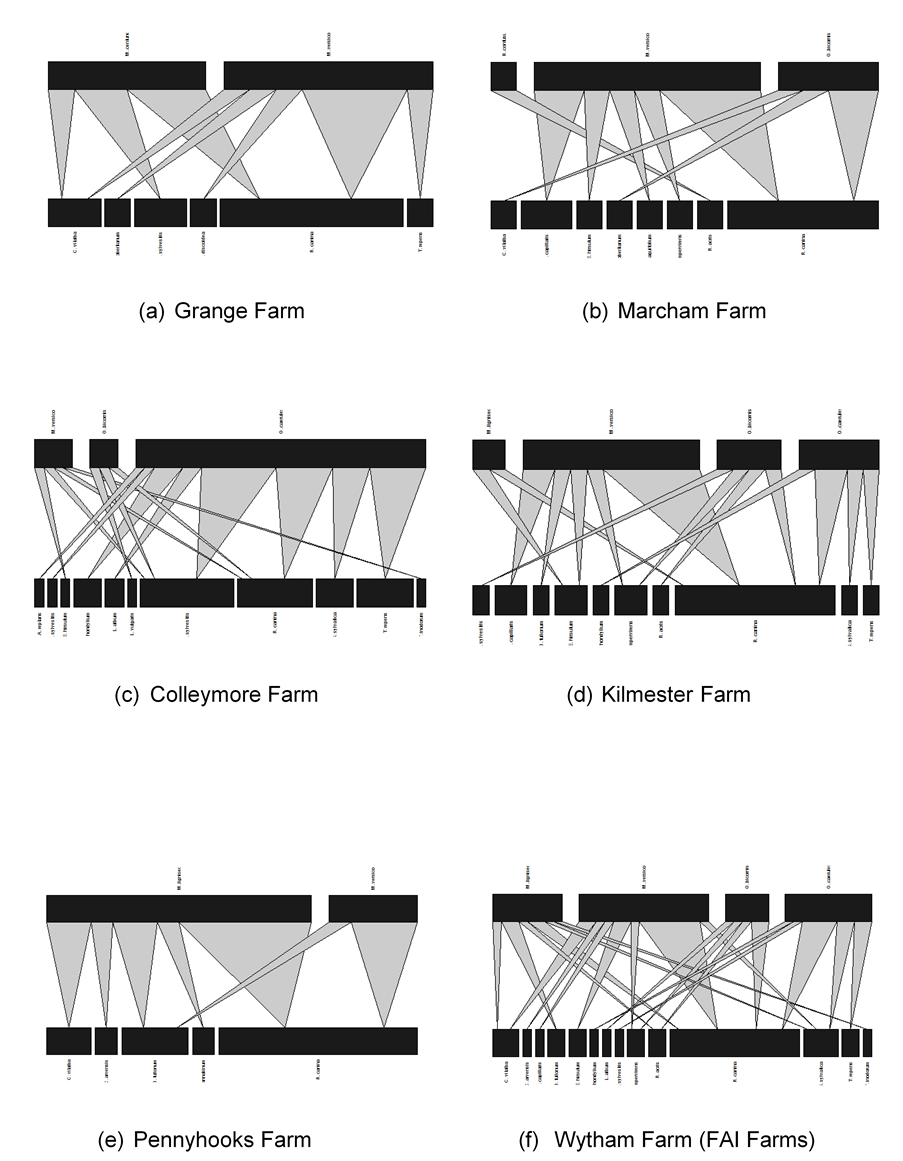


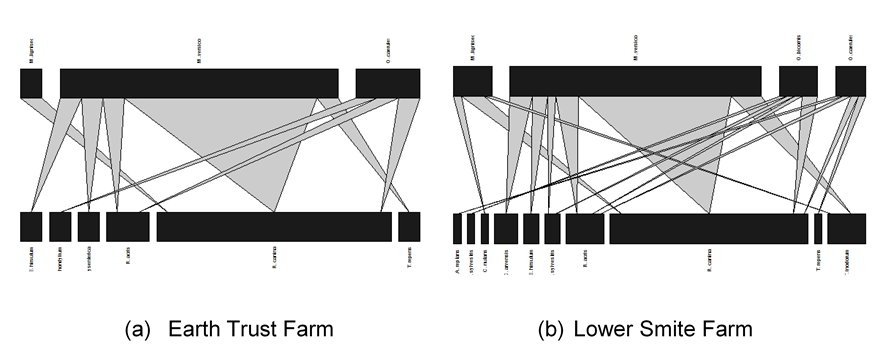


**Figure 2 (a-n)** Pollen transport network for all farms. The lower bars represent the plant species and the upper bars represent bee species. Linkage width indicates the fraction of pollen samples from that bee species in which the plant species occurred. The length of the bars for the bee species represents the number of pollen samples sequenced for each species. The length of the bars for plant species represents the frequency of occurrence of each species within the pollen samples
