# Supplementary material for: Flower preferences and pollen transport networks for cavity‐nesting solitary bees: Implications for the design of agri‐environment schemes
Source: Ecol Evol. 2018 Jul 7;8(15):7574–87. doi: 10.1002/ece3.4234 (PMC6106195; doi:10.1002/ece3.4234)
Supplement: Supplementary file 2 [file ECE3-8-7574-s002.docx]

**Flower preferences and pollen transport networks for cavity nesting solitary bees: implications for the design of agri-environment schemes**

**^1^ Catherine E. A. Gresty (corresponding author)**

76 Barrowgate Road, Chiswick, London W4 4QU

**^2^ Elizabeth Clare**

School of Biological and Chemical Sciences, Queen Mary University of London, Mile End Rd., London, E1 4NS, United Kingdom

**^3^ Dion S. Devey**

Royal Botanic Gardens, Kew, Richmond, Surrey TW9 3AE, United Kingdom

**^4^ Robyn S. Cowan**

Royal Botanic Gardens, Kew, Richmond, Surrey TW9 3AE, United Kingdom

**^5^ Laszlo Csiba**

Royal Botanic Gardens, Kew, Richmond, Surrey TW9 3AE, United Kingdom

**^6^ Panagiota Malakasi**

Royal Botanic Gardens, Kew, Richmond, Surrey TW9 3AE, United Kingdom

**^7^ Owen T. Lewis**

Department of Zoology, New Radcliffe House, Radcliffe Observatory Quarter, 6GG, Woodstock Rd, Oxford OX2

**^8^ Katherine J. Willis**

Department of Zoology, New Radcliffe House, Radcliffe Observatory Quarter, 6GG, Woodstock Rd, Oxford OX2; Royal Botanic Gardens, Kew, Richmond, Surrey TW9 3AE, United Kingdom

**Supplementary Information**

**Table 1** Summary of interventions to enhance foraging resource availability for insect pollinators under the UK Countryside Stewardship Wild Pollinator and Farm Wildlife Package (Natural England, 2016). The specific codes for each option are included in the table as these are cited commonly in the Countryside Stewardship handbooks and wider literature.

| **Options available for implementation by farmers to provide nectar and pollen sources for insect pollinators** |
| --- |
| AB1 Nectar flower mix |
| AB8 Flower-rich margins and plots |
| AB15 Two-year sown legume fallow |
| AB16 Autumn sown Bumblebird mix |
| AB11 Cultivated areas for arable plants |
| BE3 Management of hedgerows |
| GS1 Take field corners out of management |
| GS2 Permanent grassland with very low inputs |
| GS4 Legume and herb-rich swards |
| OP4 Multi-species ley |
| WD3 Woodland edges on arable land |
| WT2 Buffering in-field ponds and ditches on arable land |

**Table 2** Study farms listed according to their level of agri-environment agreement. The farms are ranked according to the level of agreement, from farms under no Environmental Stewardship scheme to those showcasing Higher Level schemes.

| **Farm** | **Location** | **Agri-Environment Level** | **Farm Size (m^2^)** |
| --- | --- | --- | --- |
| 1. Heath Farm | 52°10’32”N 1°39’59”W | No Environmental Stewardship agreement | 1262404 |
| 2. Wincott Farm | 52°08’28”N 1°43’50”W | No Environmental Stewardship agreement | 782625 |
| 3. Whitchurch Farm | 52°08’03”N 1°40’37”W | No Environmental Stewardship agreement | 1185310 |
| 4. Stepstones Farm | 52°06’29”N 1°37’42”W | No Environmental Stewardship agreement | 636314 |
| 5. Bellingdon Farm | 51°44’46”N 0°38’51”W | Entry Level Stewardship | 383767 |
| 6. Middle Farm | 51°59’16”N 1°06’52”W | Entry Level Stewardship | 1982602 |
| 7. Old Farm Dorn | 52°00’15”N 1°42’19”W | Entry Level Stewardship | 1031550 |
| 8. Daylesford | 51°55’42”N 1°38’25”W | Organic Entry Level Stewardship | 7933978 |
| 9. Grange Farm | 51°30’23”N 2°19’49”W | Higher Level Stewardship | 3094816 |
| 10. East Hanney | 51°38’06”N 1°23’14”W | Higher Level Stewardship | 1897862 |
| 11. Marcham | 51°39’56”N 1°20’37”W | Higher Level Stewardship | 4102809 |
| 12. Rotherfield Park | 51°19’57”N 0°37’45”W | Higher Level Stewardship | 16259444 |
| 13. Colleymore Farm | 51°38’30”N 1°38’30”W | Organic Higher Level Stewardship | 2824027 |
| 14. Kilmester Farm | 51°40’39”N 1°38’22”W | Organic Higher Level Stewardship | 1613061 |
| 15. Pennyhooks Farm | 51°36’31”N 1°39’36”W | Organic Higher Level Stewardship | 334842 |
| 16. Wytham Farm | 51°46’58”N 1°18’57”W | Organic Higher Level Stewardship | 4246974 |
| 17. Earth Trust | 51°37’50”N 1°11’21”W | Higher Level Stewardship, Earth Trust Management | 5039674 |
| 18. Greystones Farm | 51°53’08”N 1°45’05”W | Organic Higher Level Stewardship  Gloucester Wildlife Trust, SSSI | 666743 |
| 19. Lower Smite | 52°13’40”N 2°09’27”W | Higher Level Stewardship  Worcester Wildlife Trust, SSSI | 623146 |

**Table 3** Network metrics for the 14 farms where the networks were of sufficient size for metric computation. Pollen sample size refers to the number of pollen samples available for each farm

| **Farm** | **Stewardship** | **Pollen Sample Size** | **Connectance** | **Link Density** | **Generality (bees)** | **Nestedness** |
| --- | --- | --- | --- | --- | --- | --- |
| Heath Farm | None | 2 | 0.750 | 1.667 | 1.667 | NA |
| Wincott | None | 2 | 0.625 | 2.000 | 2.600 | -0.174 |
| Bellingdon | EL | 8 | 0.583 | 3.481 | 4.859 | 0.609 |
| Daylesford | EL | 5 | 0.625 | 1.916 | 2.396 | -0.204 |
| Old Farm Dorn | EL | 10 | 0.625 | 2.888 | 4.336 | -0.307 |
| Colleymore | HL | 10 | 0.455 | 3.718 | 5.971 | 0.489 |
| Grange Farm | HL | 7 | 0.667 | 2.548 | 3.464 | 0.288 |
| Kilmester | HL | 10 | 0.375 | 2.836 | 3.595 | 0.207 |
| Lower Smite | HL | 27 | 0.425 | 2.724 | 3.522 | 0.273 |
| Earth Trust | HL | 11 | 0.500 | 2.205 | 2.760 | 0.742 |
| Marcham | HL | 8 | 0.375 | 2.467 | 3.558 | NA |
| Fai Farms | HL | 15 | 0.393 | 3.582 | 4.820 | 0.079 |
| Pennyhooks | HL | 9 | 0.700 | 2.511 | 3.355 | 0.900 |
| Rotherfield | HL | 9 | 0.542 | 3.348 | 4.698 | 0.769 |

**Table 4** One-way ANOVA results: comparison of network metrics between farms under low agri-environment management (No Stewardship & Entry Level Stewardship farms) and high agri-environment management (Higher Level Stewardship Farms)

| **Model Type** | **Network Metric** | **Predictor** |  | **df** | **F** | **P** |
| --- | --- | --- | --- | --- | --- | --- |
| One-way ANOVA | Connectance | Agri-environment management level: Low or High |  | 2,11 | 3.37 | 0.07 |
|  | Generality (bees) | Agri-environment management level: Low or High |  | 2,11 | 2.66 | 0.11 |
|  | Link Density | Agri-environment management level: Low or High |  | 2,11 | 2.76 | 0.11 |
|  | Nestedness | Agri-environment management level: Low or High |  | 2,9 | 2.61 | 0.13 |

**Table 5** Presented are statistics of the best minimal adequate Generalized Linear Models. Model selection was based on AIC values, and ΔAIC indicates the difference between the best and next best model and ΔAIC compared to intercept only model indicates the difference between the best fit model and intercept only model. Coefficients of determination, *D*^2^ (the amount of deviance accounted for by the model) are provided – these were calculated using the “modEvA” package in R (Barbosa, et al., 2016). The full set of explanatory variables included in the full models for models were: floral-unit abundance, the proportion of natural habitat, the number of pollen samples available and the number of bee samples from which pollen samples were taken for each farm.

| **Model** | **Response Variable** | **Explanatory Variables** | **df** | **F** | **P** |
| --- | --- | --- | --- | --- | --- |
|  | Best model, AIC = -39.56, Adj. D2 =0.834, ΔAIC = 1.23, ΔAIC compared to intercept only model = 24.3 | | | | |
| 1 | Connectance | Floral unit abundance | 1,12 | 2.497 | 0.007 |
|  |  | Plant species richness | 1,11 | 23.394 | 0.004 |
|  |  | Bee species richness | 1,10 | 51.167 | <0.001 |
|  | Best model, AIC = 29, Adj. D2 =0.077, ΔAIC =0.35, ΔAIC compared to intercept only model = 1.46 | | | | |
| 2 | Link density | Bee species richness | 1,12 | 3.369 | 0.0913 |
|  |  |  |  |  |  |
|  | Best model, AIC =45.82, Adj. D2 =0.0388ΔAIC = 0.97, ΔAIC compared to intercept only model = 0.89 | | | | |
| 3 | Generality (bees) | Plant species richness | 1,12 | 2.754 | 0.123 |
|  |  |  |  |  |  |
|  | Best model, AIC = 0.74, Adj. D2 = 0.694, ΔAIC = 1.73, ΔAIC compared to intercept only model = 14.62 | | | | |
| 5 | Nestedness | Floral unit abundance | 1,10 | 29.953 | <0.001 |
|  |  |  |  |  |  |
|  | Best model, AIC = 73.19, Adj. D2 = 0.453, ΔAIC = 0.23, ΔAIC compared to intercept only model = 2.1 | | | | |
| 6 | Number of plant species visited within farm networks | Floral unit abundance | 1,13 | 2.685 | 0.222 |
|  |  | Plant species richness | 1,12 | 5.098 | 0.175 |
|  |  | Bee species richness | 1,11 | 9.356 | 0.011 |

**Table 6** This table provides the full pollen DNA output data for this study. Listed are the pollen sample IDs (this corresponds to the nest box and bamboo shoot the pollen was extracted from), the farm the pollen sample was taken from, the bee species the pollen sample came from and the plant species identified from the DNA analysis.

| **Sample ID** | **Farm** | **Bee Species** | **Plant Species** |
| --- | --- | --- | --- |
| B9-1 | Bellingdon | *Hylaeus confusus* | *Malva sylvestris* |
| B9-1 | Bellingdon | *Hylaeus confusus* | *Rosa canina* |
| B9-1 | Bellingdon | *Hylaeus confusus* | *Stachys sylvatica* |
| B9-4 | Bellingdon | *Megachile ligniseca* | *Malva sylvestris* |
| B9-4 | Bellingdon | *Megachile ligniseca* | *Rosa canina* |
| B9-8 | Bellingdon | *Megachile ligniseca* | *Crepis capillaris* |
| B9-8 | Bellingdon | *Megachile ligniseca* | *Rosa canina* |
| B9-8 | Bellingdon | *Megachile ligniseca* | *Tripleurospermum inodorum* |
| B14-2 | Bellingdon | *Megachile versicolor* | *Ranunculus acris* |
| B14-2 | Bellingdon | *Megachile versicolor* | *Rosa canina* |
| B9-2 | Bellingdon | *Megachile versicolor* | *Crepis capillaris* |
| B9-2 | Bellingdon | *Megachile versicolor* | *Dipsacus fullonum* |
| B9-2 | Bellingdon | *Megachile versicolor* | *Malva sylvestris* |
| B9-2 | Bellingdon | *Megachile versicolor* | *Rosa canina* |
| B9-2 | Bellingdon | *Megachile versicolor* | *Tripleurospermum inodorum* |
| B9-5 | Bellingdon | *Megachile versicolor* | *Malva sylvestris* |
| B9-5 | Bellingdon | *Megachile versicolor* | *Rosa canina* |
| B9-6 | Bellingdon | *Megachile versicolor* | *Convolvulus arvensis* |
| B9-6 | Bellingdon | *Megachile versicolor* | *Crepis capillaris* |
| B9-6 | Bellingdon | *Megachile versicolor* | *Malva sylvestris* |
| B9-6 | Bellingdon | *Megachile versicolor* | *Rosa canina* |
| B9-7 | Bellingdon | *Megachile versicolor* | *Rosa canina* |
| C14-6 | Colleymore | *Megachile versicolor* | *Epilobium hirsutum* |
| C14-6 | Colleymore | *Megachile versicolor* | *Malva sylvestris* |
| C14-6 | Colleymore | *Megachile versicolor* | *Rosa canina* |
| C14-6 | Colleymore | *Megachile versicolor* | *Tripleurospermum inodorum* |
| C14-1 | Colleymore | *Osmia bicornis* | *Lysimachia vulgaris* |
| C14-1 | Colleymore | *Osmia bicornis* | *Malva sylvestris* |
| C14-1 | Colleymore | *Osmia bicornis* | *Rosa canina* |
| C11-2 | Colleymore | *Osmia caerulescens* | *Malva sylvestris* |
| C11-2 | Colleymore | *Osmia caerulescens* | *Rosa canina* |
| C11-2 | Colleymore | *Osmia caerulescens* | *Stachys sylvatica* |
| C11-2 | Colleymore | *Osmia caerulescens* | *Trifolium repens* |
| C11-3 | Colleymore | *Osmia caerulescens* | *Lamium album* |
| C11-3 | Colleymore | *Osmia caerulescens* | *Malva sylvestris* |
| C11-3 | Colleymore | *Osmia caerulescens* | *Rosa canina* |
| C11-3 | Colleymore | *Osmia caerulescens* | *Stachys sylvatica* |
| C11-3 | Colleymore | *Osmia caerulescens* | *Trifolium repens* |
| C11-4 | Colleymore | *Osmia caerulescens* | *Malva sylvestris* |
| C11-4 | Colleymore | *Osmia caerulescens* | *Rosa canina* |
| C14-2 | Colleymore | *Osmia caerulescens* | *Ajuga reptans* |
| C14-2 | Colleymore | *Osmia caerulescens* | *Anthriscus sylvestris* |
| C14-2 | Colleymore | *Osmia caerulescens* | *Heracleum sphondylium* |
| C14-2 | Colleymore | *Osmia caerulescens* | *Lamium album* |
| C14-2 | Colleymore | *Osmia caerulescens* | *Malva sylvestris* |
| C14-2 | Colleymore | *Osmia caerulescens* | *Rosa canina* |
| C14-2 | Colleymore | *Osmia caerulescens* | *Trifolium repens* |
| C14-4 | Colleymore | *Osmia caerulescens* | *Malva sylvestris* |
| C14-4 | Colleymore | *Osmia caerulescens* | *Rosa canina* |
| C14-4 | Colleymore | *Osmia caerulescens* | *Trifolium repens* |
| C14-7 | Colleymore | *Osmia caerulescens* | *Heracleum sphondylium* |
| C14-7 | Colleymore | *Osmia caerulescens* | *Malva sylvestris* |
| C14-7 | Colleymore | *Osmia caerulescens* | *Stachys sylvatica* |
| C14-7 | Colleymore | *Osmia caerulescens* | *Trifolium repens* |
| C14-8 | Colleymore | *Osmia caerulescens* | *Heracleum sphondylium* |
| C14-8 | Colleymore | *Osmia caerulescens* | *Malva sylvestris* |
| C14-8 | Colleymore | *Osmia caerulescens* | *Rosa canina* |
| C14-8 | Colleymore | *Osmia caerulescens* | *Stachys sylvatica* |
| C14-8 | Colleymore | *Osmia caerulescens* | *Trifolium repens* |
| C4-5 | Colleymore | *Osmia caerulescens* | *Malva sylvestris* |
| D14-1 | Daylesford | *Megachile ligniseca* | *Dipsacus fullonum* |
| D14-1 | Daylesford | *Megachile ligniseca* | *Rosa canina* |
| D14-2 | Daylesford | *Megachile ligniseca* | *Dipsacus fullonum* |
| D14-2 | Daylesford | *Megachile ligniseca* | *Rosa canina* |
| D14-2 | Daylesford | *Megachile ligniseca* | *Trifolium repens* |
| D13-1 | Daylesford | *Megachile versicolor* | *Epilobium hirsutum* |
| D13-1 | Daylesford | *Megachile versicolor* | *Rosa canina* |
| D13-2 | Daylesford | *Megachile versicolor* | *Epilobium hirsutum* |
| D13-2 | Daylesford | *Megachile versicolor* | *Rosa canina* |
| D13-3 | Daylesford | *Megachile versicolor* | *Epilobium hirsutum* |
| D13-3 | Daylesford | *Megachile versicolor* | *Rosa canina* |
| LW3-2 | Earth Trust | *Megachile ligniseca* | *Rosa canina* |
| LW10-6 | Earth Trust | *Megachile versicolor* | *Rosa canina* |
| LW14-1 | Earth Trust | *Megachile versicolor* | *Rosa canina* |
| LW14-3 | Earth Trust | *Megachile versicolor* | *Pulicaria dysenterica* |
| LW14-3 | Earth Trust | *Megachile versicolor* | *Rosa canina* |
| LW14-3 | Earth Trust | *Megachile versicolor* | *Trifolium repens* |
| LW3-1 | Earth Trust | *Megachile versicolor* | *Rosa canina* |
| LW9-1 | Earth Trust | *Megachile versicolor* | *Epilobium hirsutum* |
| LW9-1 | Earth Trust | *Megachile versicolor* | *Rosa canina* |
| LW9-12 | Earth Trust | *Megachile versicolor* | *Rosa canina* |
| LW9-3 | Earth Trust | *Megachile versicolor* | *Rosa canina* |
| LW9-7 | Earth Trust | *Megachile versicolor* | *Rosa canina* |
| LW9-8 | Earth Trust | *Megachile versicolor* | *Ranunculus acris* |
| LW9-8 | Earth Trust | *Megachile versicolor* | *Rosa canina* |
| LW11-2 | Earth Trust | *Osmia caerulescens* | *Heracleum sphondylium* |
| LW11-2 | Earth Trust | *Osmia caerulescens* | *Ranunculus acris* |
| LW11-2 | Earth Trust | *Osmia caerulescens* | *Rosa canina* |
| O14-13 | Fai Farms | *Megachile ligniseca* | *Dipsacus fullonum* |
| O14-13 | Fai Farms | *Megachile ligniseca* | *Rosa canina* |
| O14-13 | Fai Farms | *Megachile ligniseca* | *Stachys sylvatica* |
| O14-18 | Fai Farms | *Megachile ligniseca* | *Clematis vitalba* |
| O14-18 | Fai Farms | *Megachile ligniseca* | *Dipsacus fullonum* |
| O14-18 | Fai Farms | *Megachile ligniseca* | *Ranunculus acris* |
| O14-18 | Fai Farms | *Megachile ligniseca* | *Rosa canina* |
| O14-18 | Fai Farms | *Megachile ligniseca* | *Tripleurospermum inodorum* |
| O13-2 | Fai Farms | *Megachile versicolor* | *Pentaglottis sempervirens* |
| O13-2 | Fai Farms | *Megachile versicolor* | *Rosa canina* |
| O14-16 | Fai Farms | *Megachile versicolor* | *Epilobium hirsutum* |
| O14-16 | Fai Farms | *Megachile versicolor* | *Rosa canina* |
| O14-17 | Fai Farms | *Megachile versicolor* | *Epilobium hirsutum* |
| O14-17 | Fai Farms | *Megachile versicolor* | *Rosa canina* |
| O14-17 | Fai Farms | *Megachile versicolor* | *Stachys sylvatica* |
| O14-3 | Fai Farms | *Megachile versicolor* | *Convolvulus arvensis* |
| O14-3 | Fai Farms | *Megachile versicolor* | *Rosa canina* |
| O14-5 | Fai Farms | *Megachile versicolor* | *Clematis vitalba* |
| O14-5 | Fai Farms | *Megachile versicolor* | *Rosa canina* |
| O14-9 | Fai Farms | *Megachile versicolor* | *Rosa canina* |
| O15-1 | Fai Farms | *Megachile versicolor* | *Clematis vitalba* |
| O15-1 | Fai Farms | *Megachile versicolor* | *Crepis capillaris* |
| O15-1 | Fai Farms | *Megachile versicolor* | *Rosa canina* |
| O6-1 | Fai Farms | *Osmia bicornis* | *Lamium album* |
| O6-1 | Fai Farms | *Osmia bicornis* | *Rosa canina* |
| O6-3 | Fai Farms | *Osmia bicornis* | *Pentaglottis sempervirens* |
| O6-3 | Fai Farms | *Osmia bicornis* | *Ranunculus acris* |
| O6-3 | Fai Farms | *Osmia bicornis* | *Rosa canina* |
| O14-15 | Fai Farms | *Osmia caerulescens* | *Rosa canina* |
| O14-15 | Fai Farms | *Osmia caerulescens* | *Stachys sylvatica* |
| O14-15 | Fai Farms | *Osmia caerulescens* | *Trifolium repens* |
| O14-4 | Fai Farms | *Osmia caerulescens* | *Rosa canina* |
| O14-4B | Fai Farms | *Osmia caerulescens* | *Rosa canina* |
| O14-7 | Fai Farms | *Osmia caerulescens* | *Heracleum sphondylium* |
| O14-7 | Fai Farms | *Osmia caerulescens* | *Malva sylvestris* |
| O14-7 | Fai Farms | *Osmia caerulescens* | *Rosa canina* |
| O14-7 | Fai Farms | *Osmia caerulescens* | *Stachys sylvatica* |
| O14-7 | Fai Farms | *Osmia caerulescens* | *Trifolium repens* |
| GF12-3 | Grange Farm | *Megachile centuncularis* | *Malva sylvestris* |
| GF12-3 | Grange Farm | *Megachile centuncularis* | *Rosa canina* |
| GF12-5 | Grange Farm | *Megachile centuncularis* | *Clematis vitalba* |
| GF12-5 | Grange Farm | *Megachile centuncularis* | *Malva sylvestris* |
| GF12-5 | Grange Farm | *Megachile centuncularis* | *Rosa canina* |
| GF13-1 | Grange Farm | *Megachile centuncularis* | *Rosa canina* |
| GF14-1 | Grange Farm | *Megachile versicolor* | *Rosa canina* |
| GF14-2 | Grange Farm | *Megachile versicolor* | *Geranium robertianum* |
| GF14-2 | Grange Farm | *Megachile versicolor* | *Rosa canina* |
| GF14-3 | Grange Farm | *Megachile versicolor* | *Rosa canina* |
| GF14-3 | Grange Farm | *Megachile versicolor* | *Trifolium repens* |
| GF5-4 | Grange Farm | *Megachile versicolor* | *Clematis vitalba* |
| GF5-4 | Grange Farm | *Megachile versicolor* | *Matricaria discoidea* |
| GF5-4 | Grange Farm | *Megachile versicolor* | *Rosa canina* |
| H14-2 | Heath Farm | *Megachile versicolor* | *Clematis vitalba* |
| H14-2 | Heath Farm | *Megachile versicolor* | *Rosa canina* |
| H8-2 | Heath Farm | *Osmia caerulescens* | *Rosa canina* |
| K14-4 | Kilmester | *Megachile ligniseca* | *Epilobium hirsutum* |
| K14-4 | Kilmester | *Megachile ligniseca* | *Rosa canina* |
| K11-1 | Kilmester | *Megachile versicolor* | *Rosa canina* |
| K11-3 | Kilmester | *Megachile versicolor* | *Rosa canina* |
| K11-4 | Kilmester | *Megachile versicolor* | *Crepis capillaris* |
| K11-4 | Kilmester | *Megachile versicolor* | *Pentaglottis sempervirens* |
| K11-4 | Kilmester | *Megachile versicolor* | *Rosa canina* |
| K14-3 | Kilmester | *Megachile versicolor* | *Rosa canina* |
| K7-2 | Kilmester | *Megachile versicolor* | *Rosa canina* |
| K8-2 | Kilmester | *Megachile versicolor* | *Crepis capillaris* |
| K8-2 | Kilmester | *Megachile versicolor* | *Dipsacus fullonum* |
| K8-2 | Kilmester | *Megachile versicolor* | *Epilobium hirsutum* |
| K8-2 | Kilmester | *Megachile versicolor* | *Rosa canina* |
| K11-2 | Kilmester | *Osmia bicornis* | *Anthriscus sylvestris* |
| K11-2 | Kilmester | *Osmia bicornis* | *Pentaglottis sempervirens* |
| K11-2 | Kilmester | *Osmia bicornis* | *Ranunculus acris* |
| K11-2 | Kilmester | *Osmia bicornis* | *Rosa canina* |
| K13-2 | Kilmester | *Osmia caerulescens* | *Heracleum sphondylium* |
| K13-2 | Kilmester | *Osmia caerulescens* | *Rosa canina* |
| K13-2 | Kilmester | *Osmia caerulescens* | *Stachys sylvatica* |
| K13-2 | Kilmester | *Osmia caerulescens* | *Trifolium repens* |
| K14-2 | Kilmester | *Osmia caerulescens* | *Rosa canina* |
| LS13-1 | Lower Smite | *Megachile ligniseca* | *Carduus nutan* |
| LS13-1 | Lower Smite | *Megachile ligniseca* | *Rosa canina* |
| LS9-2 | Lower Smite | *Megachile ligniseca* | *Rosa canina* |
| LS9-2 | Lower Smite | *Megachile ligniseca* | *Tripleurospermum inodorum* |
| LS9-4 | Lower Smite | *Megachile ligniseca* | *Rosa canina* |
| LS11-1 | Lower Smite | *Megachile versicolor* | *Epilobium hirsutum* |
| LS11-1 | Lower Smite | *Megachile versicolor* | *Rosa canina* |
| LS11-3 | Lower Smite | *Megachile versicolor* | *Rosa canina* |
| LS11-4 | Lower Smite | *Megachile versicolor* | *Rosa canina* |
| LS11-5 | Lower Smite | *Megachile versicolor* | *Rosa canina* |
| LS11-6 | Lower Smite | *Megachile versicolor* | *Rosa canina* |
| LS11-8 | Lower Smite | *Megachile versicolor* | *Malva sylvestris* |
| LS11-8 | Lower Smite | *Megachile versicolor* | *Rosa canina* |
| LS13-2 | Lower Smite | *Megachile versicolor* | *Rosa canina* |
| LS14-1 | Lower Smite | *Megachile versicolor* | *Rosa canina* |
| LS14-2 | Lower Smite | *Megachile versicolor* | *Rosa canina* |
| LS14-4 | Lower Smite | *Megachile versicolor* | *Rosa canina* |
| LS14-5 | Lower Smite | *Megachile versicolor* | *Rosa canina* |
| LS14-5 | Lower Smite | *Megachile versicolor* | *Tripleurospermum inodorum* |
| LS14-6 | Lower Smite | *Megachile versicolor* | *Convolvulus arvensis* |
| LS14-6 | Lower Smite | *Megachile versicolor* | *Epilobium hirsutum* |
| LS14-6 | Lower Smite | *Megachile versicolor* | *Ranunculus acris* |
| LS14-6 | Lower Smite | *Megachile versicolor* | *Rosa canina* |
| LS14-6 | Lower Smite | *Megachile versicolor* | *Tripleurospermum inodorum* |
| LS14-7 | Lower Smite | *Megachile versicolor* | *Rosa canina* |
| LS2-3B | Lower Smite | *Megachile versicolor* | *Rosa canina* |
| LS5-4 | Lower Smite | *Megachile versicolor* | *Convolvulus arvensis* |
| LS5-5 | Lower Smite | *Megachile versicolor* | *Ranunculus acris* |
| LS5-5 | Lower Smite | *Megachile versicolor* | *Rosa canina* |
| LS9-1 | Lower Smite | *Megachile versicolor* | *Convolvulus arvensis* |
| LS9-1 | Lower Smite | *Megachile versicolor* | *Rosa canina* |
| LS9-11 | Lower Smite | *Megachile versicolor* | *Rosa canina* |
| LS9-3 | Lower Smite | *Megachile versicolor* | *Rosa canina* |
| LS9-3 | Lower Smite | *Megachile versicolor* | *Tripleurospermum inodorum* |
| LS9-5 | Lower Smite | *Megachile versicolor* | *Rosa canina* |
| LS9-8 | Lower Smite | *Megachile versicolor* | *Ranunculus acris* |
| LS9-8 | Lower Smite | *Megachile versicolor* | *Rosa canina* |
| LS9-8 | Lower Smite | *Megachile versicolor* | *Tripleurospermum inodorum* |
| LS15-1 | Lower Smite | *Osmia bicornis* | *Anthriscus sylvestris* |
| LS15-1 | Lower Smite | *Osmia bicornis* | *Malva sylvestris* |
| LS15-1 | Lower Smite | *Osmia bicornis* | *Ranunculus acris* |
| LS15-1 | Lower Smite | *Osmia bicornis* | *Rosa canina* |
| LS15-2 | Lower Smite | *Osmia bicornis* | *Rosa canina* |
| LS2-7 | Lower Smite | *Osmia caerulescens* | *Ajuga reptans* |
| LS2-7 | Lower Smite | *Osmia caerulescens* | *Ranunculus acris* |
| LS2-7 | Lower Smite | *Osmia caerulescens* | *Rosa canina* |
| LS2-7 | Lower Smite | *Osmia caerulescens* | *Trifolium repens* |
| M3-1 | Marcham | *Hylaeus confusus* | *Ranunculus acris* |
| M1-1 | Marcham | *Megachile versicolor* | *Rosa canina* |
| M10-3 | Marcham | *Megachile versicolor* | *Ilex aquifolium* |
| M10-3 | Marcham | *Megachile versicolor* | *Pentaglottis sempervirens* |
| M10-3 | Marcham | *Megachile versicolor* | *Rosa canina* |
| M15-11 | Marcham | *Megachile versicolor* | *Crepis capillaris* |
| M15-11 | Marcham | *Megachile versicolor* | *Rosa canina* |
| M15-6 | Marcham | *Megachile versicolor* | *Crepis capillaris* |
| M15-6 | Marcham | *Megachile versicolor* | *Epilobium hirsutum* |
| M15-6 | Marcham | *Megachile versicolor* | *Rosa canina* |
| M3-2 | Marcham | *Osmia bicornis* | *Clematis vitalba* |
| M3-5 | Marcham | *Osmia bicornis* | *Geranium robertianum* |
| M3-5 | Marcham | *Osmia bicornis* | *Rosa canina* |
| M9-3 | Marcham | *Osmia bicornis* | *Rosa canina* |
| OD3-1 | Old Farm Dorn | *Megachile versicolor* | *Rosa canina* |
| OD3-1 | Old Farm Dorn | *Megachile versicolor* | *Tripleurospermum inodorum* |
| OD3-2 | Old Farm Dorn | *Megachile versicolor* | *Rosa canina* |
| OD6-1 | Old Farm Dorn | *Megachile versicolor* | *Malva sylvestris* |
| OD6-1 | Old Farm Dorn | *Megachile versicolor* | *Rosa canina* |
| OD6-1 | Old Farm Dorn | *Megachile versicolor* | *Tripleurospermum inodorum* |
| OD6-2 | Old Farm Dorn | *Megachile versicolor* | *Malva sylvestris* |
| OD6-2 | Old Farm Dorn | *Megachile versicolor* | *Tripleurospermum inodorum* |
| OD6-3 | Old Farm Dorn | *Megachile versicolor* | *Epilobium hirsutum* |
| OD6-3 | Old Farm Dorn | *Megachile versicolor* | *Malva sylvestris* |
| OD6-3 | Old Farm Dorn | *Megachile versicolor* | *Ranunculus acris* |
| OD6-3 | Old Farm Dorn | *Megachile versicolor* | *Rosa canina* |
| OD6-3 | Old Farm Dorn | *Megachile versicolor* | *Tripleurospermum inodorum* |
| OD6-4 | Old Farm Dorn | *Megachile versicolor* | *Rosa canina* |
| OD7-1 | Old Farm Dorn | *Megachile versicolor* | *Rosa canina* |
| OD2-4 | Old Farm Dorn | *Osmia caerulescens* | *Rosa canina* |
| OD3-3 | Old Farm Dorn | *Osmia caerulescens* | *Heracleum sphondylium* |
| OD3-3 | Old Farm Dorn | *Osmia caerulescens* | *Malva sylvestris* |
| OD3-3 | Old Farm Dorn | *Osmia caerulescens* | *Rosa canina* |
| OD3-3 | Old Farm Dorn | *Osmia caerulescens* | *Stachys sylvatica* |
| OD3-3 | Old Farm Dorn | *Osmia caerulescens* | *Trifolium repens* |
| OD3-5 | Old Farm Dorn | *Osmia caerulescens* | *Heracleum sphondylium* |
| OD3-5 | Old Farm Dorn | *Osmia caerulescens* | *Rosa canina* |
| OD3-5 | Old Farm Dorn | *Osmia caerulescens* | *Stachys sylvatica* |
| OD3-5 | Old Farm Dorn | *Osmia caerulescens* | *Trifolium repens* |
| P12-1 | Pennyhooks | *Megachile ligniseca* | *Rosa canina* |
| P14-1 | Pennyhooks | *Megachile ligniseca* | *Rosa canina* |
| P14-2 | Pennyhooks | *Megachile ligniseca* | *Dipsacus fullonum* |
| P14-2 | Pennyhooks | *Megachile ligniseca* | *Rosa canina* |
| P14-4 | Pennyhooks | *Megachile ligniseca* | *Rosa canina* |
| P4-1 | Pennyhooks | *Megachile ligniseca* | *Clematis vitalba* |
| P4-1 | Pennyhooks | *Megachile ligniseca* | *Convolvulus arvensis* |
| P4-1 | Pennyhooks | *Megachile ligniseca* | *Dipsacus fullonum* |
| P4-1 | Pennyhooks | *Megachile ligniseca* | *Rosa canina* |
| P4-2 | Pennyhooks | *Megachile ligniseca* | *Clematis vitalba* |
| P4-2 | Pennyhooks | *Megachile ligniseca* | *Eupatorium cannabinum* |
| P4-2 | Pennyhooks | *Megachile ligniseca* | *Rosa canina* |
| P11-1 | Pennyhooks | *Megachile versicolor* | *Rosa canina* |
| P11-2 | Pennyhooks | *Megachile versicolor* | *Dipsacus fullonum* |
| P11-2 | Pennyhooks | *Megachile versicolor* | *Rosa canina* |
| P14-3 | Pennyhooks | *Megachile versicolor* | *Rosa canina* |
| R8-1 | Rotherfield | *Megachile ligniseca* | *Rosa canina* |
| R8-1 | Rotherfield | *Megachile ligniseca* | *Tripleurospermum inodorum* |
| R12-3 | Rotherfield | *Megachile versicolor* | *Crepis capillaris* |
| R12-3 | Rotherfield | *Megachile versicolor* | *Malva sylvestris* |
| R12-3 | Rotherfield | *Megachile versicolor* | *Rosa canina* |
| R12-3 | Rotherfield | *Megachile versicolor* | *Tripleurospermum inodorum* |
| R12-5 | Rotherfield | *Megachile versicolor* | *Rosa canina* |
| R12-6 | Rotherfield | *Megachile versicolor* | *Rosa canina* |
| R12-6 | Rotherfield | *Megachile versicolor* | *Tripleurospermum inodorum* |
| R13-1 | Rotherfield | *Megachile versicolor* | *Clematis vitalba* |
| R13-1 | Rotherfield | *Megachile versicolor* | *Heracleum sphondylium* |
| R13-1 | Rotherfield | *Megachile versicolor* | *Malva sylvestris* |
| R13-1 | Rotherfield | *Megachile versicolor* | *Ranunculus acris* |
| R13-1 | Rotherfield | *Megachile versicolor* | *Rosa canina* |
| R13-1 | Rotherfield | *Megachile versicolor* | *Tripleurospermum inodorum* |
| R3-1 | Rotherfield | *Megachile versicolor* | *Rosa canina* |
| R8-3 | Rotherfield | *Megachile versicolor* | *Rosa canina* |
| R1-2 | Rotherfield | *Osmia caerulescens* | *Heracleum sphondylium* |
| R1-2 | Rotherfield | *Osmia caerulescens* | *Rosa canina* |
| R1-2 | Rotherfield | *Osmia caerulescens* | *Tripleurospermum inodorum* |
| R1-5 | Rotherfield | *Osmia caerulescens* | *Epilobium hirsutum* |
| R1-5 | Rotherfield | *Osmia caerulescens* | *Rosa canina* |
| WT12-3 | Whitchurch | *Osmia bicornis* | *Anthriscus sylvestris* |
| WT12-4 | Whitchurch | *Osmia bicornis* | *Anthriscus sylvestris* |
| WT12-4 | Whitchurch | *Osmia bicornis* | *Rosa canina* |
| WT12-7 | Whitchurch | *Osmia bicornis* | *Ranunculus acris* |
| WT12-7 | Whitchurch | *Osmia bicornis* | *Rosa canina* |
| WN11-2 | Wincott | *Megachile versicolor* | *Ranunculus acris* |
| WN11-2 | Wincott | *Megachile versicolor* | *Rosa canina* |
| WN15-1 | Wincott | *Osmia caerulescens* | *Anthriscus sylvestris* |
| WN15-1 | Wincott | *Osmia caerulescens* | *Rosa canina* |
| WN15-1 | Wincott | *Osmia caerulescens* | *Trifolium repens* |

**Table 7** This table lists what constituted a flower unit for each plant species recorded within this study. The flower units were categorized as a single solitary flower or capitulum (e.g. *Leucanthemum vulgare*), cyme (e.g. *Myosotis arvensis*), raceme (e.g. *Lysimachia vulgaris*), umbel (e.g. *Anthriscus sylvestris*), corymb (e.g. *Jacobea vulgaris*) or panicle (e.g. *Centranthus ruber*) as appropriate, following (Clapham, Tutin & Moore, 1987; Rose, 2016; Staces, 2010). Also included are the species authorities.

| **Plant Species List** | **Species Authority** | **Floral Unit** |
| --- | --- | --- |
| *Achillea millefolium* | L. | Corymb (of capitula) |
| *Aegopodium podagraria* | L. | Umbel |
| *Agrimonia eupatoria* | L. | Raceme |
| *Agrostemma githago* | L. | Solitary (capitulum) |
| *Ajuga reptans* | L. | Spike (of contracted cymes) |
| *Alliaria petiolata* | (M.Bieb.) Cavara & Grande | Raceme |
| *Allium ursinum* | L. | Umbel |
| *Anagallis arvensis* | (L.) U.Manns & Anderb. | Solitary |
| *Angelica sylvestris* | L. | Umbel |
| *Anthemis arvensis* | L. | Solitary (capitulum) |
| *Anthemis austriaca* | Jacq. | Solitary (capitulum) |
| *Anthriscus sylvestris* | (L.) Hoffm. | Umbel |
| *Arctium minus* | (Hill) Bernh. | Solitary (capitulum) |
| *Argyranthemum frutescens* | (L.) Sch.Bip. | Solitary (capitulum) |
| *Arum maculatum* | L. | Spike (spadix) |
| *Atropa bella-donna* | L. | Solitary |
| *Ballota nigra* | L. | Spike |
| *Bellis perennis* | L. | Solitary (capitulum) |
| *Bergenia* sp. | NA | Panicle |
| *Brassica napus* | Burnett | Raceme |
| *Buddleja* sp. | NA | Spike |
| *Calystegia sepium* | (L.) R.Br. | Solitary |
| *Calystegia silvatica* | (Kit.) Griseb. | Solitary |
| *Capsella bursa-pastoris* | (L.) Medik. | Raceme |
| *Cardamine hirsuta* | L. | Raceme |
| *Cardamine pratensis* | L. | Raceme |
| *Carduus nutans* | L. | Solitary (capitulum) |
| *Centaurea cyanus* | L. | Solitary (capitulum) |
| *Centaurea nigra* | L. | Solitary (capitulum) |
| *Centaurium erythraea* | Rafn | Solitary (capitulum) |
| *Centranthus ruber* | (L.) DC. | Panicle |
| *Cerinthe* sp. | NA | Cyme |
| *Chaenomeles sp.* | NA | Solitary |
| *Cichorium intybus* | L. | Solitary (capitulum) |
| *Cirsium arvense* | (L.) Scop. | Solitary (capitulum) |
| *Cirsium palustre* | (L.) Scop. | Solitary (capitulum) |
| *Cirsium vulgare* | (Savi) Ten. | Solitary (capitulum) |
| *Clinopodium vulgare* | L. | Cyme |
| *Conopodium majus* | (Gouan) Loret | Umbel |
| *Convolvulus arvensis* | L. | Solitary |
| *Crataegus monogyna* | Jacq. | Corymb |
| *Crepis capillaris* | (L.) Wallr. | Corymb (of capitula) |
| *Dactylorhiza fuchsii* | (Druce) Soó | Spike |
| *Dactylorhiza majalis* | (Rchb.) P.F.Hunt & Summerh. | Spike |
| *Dactylorhiza praetermissa* | (Druce) Soó | Spike |
| *Daucus carota* | L. | Umbel |
| *Digitalis purpurea* | L. | Raceme |
| *Dipsacus fullonum* | L. | Solitary (capitulum) |
| *Dipsacus fullonum* | L. | Solitary (capitulum) |
| *Echium vulgare* | L. | Panicle (of cymes) |
| *Epilobium angustifolium* | L. | Spike |
| *Epilobium angustifolium* | L. | Raceme |
| *Epilobium hirsutum* | L. | Raceme |
| *Epilobium montanum* | L. | Raceme |
| *Epilobium strictum* | Muhl. ex Spreng. | Raceme |
| *Eryngium maritimum* | L. | Solitary (capitulum) |
| *Euphorbia peplus* | L. | Umbel (of cymes) |
| *Euphorbia* sp. | L. | Umbel (of cymes) |
| *Ficaria verna* | Huds. | Solitary |
| *Filipendula ulmaria* | (L.) Maxim. | Cymose panicle |
| *Fragaria × ananassa* | (Duchesne ex Weston) Duchesne ex Rozier | Cyme |
| *Fragaria virginiana* | Mill. | Cyme |
| *Fragaria x ananassa* | J. Gay | Cyme |
| *Galium verum* | L. | Panicle |
| *Geranium molle* | L. | Cyme |
| *Geranium pratense* | L. | Cyme |
| *Geranium robertianum* | L. | Cyme |
| *Geranium* sp. | NA | Cyme |
| *Glechoma hederacea* | L. | Cyme |
| *Helianthus annuus* | L. | Solitary (capitulum) |
| *Helminthotheca echioides* | (L.) Holub. | Corymb |
| *Heracleum sphondylium* | L. | Umbel |
| *Hieracium* sp. | NA | Umbel |
| *Hyacinthoides hispanica* | (Mill.) Rothm. | Raceme |
| *Hydrangea* sp. | NA | Corymb |
| *Hypericum perforatum* | L. | Cyme |
| *Ilex aquifolium* | L. | Cyme |
| *Iris pseudacorus* | L. | Solitary |
| *Iris* sp. | NA | Solitary |
| *Jacobaea vulgaris* | P. Gaertn. | Corymb (of capitula) |
| *Knautia arvensis* | (L.) Coult. | Solitary (capitulum) |
| *Lamium album* | L. | Cyme |
| *Lamium purpureum* | L. | Cyme |
| *Lathyrus latifolius* | L. | Cyme |
| *Lavandula angustifolia* | Mill. | Spike |
| *Lavandula* sp. | NA | Spike |
| *Lepidium draba* | L. | Raceme |
| *Leucanthemum vulgare* | Lam. | Solitary |
| *Linum usitatissimum* | L. | Cyme |
| *Lithospermum arvense* | L. | Cyme |
| *Lotus corniculatus* | L. | Cyme |
| *Lunaria annua* | L. | Raceme |
| *Lychnis flos-cuculi* | (L.) Greuter & Burdet | Cyme |
| *Lycopus europaeus* | L. | Cyme |
| *Lysimachia punctata* | L. | Raceme |
| *Lysimachia vulgaris* | L. | Panicle |
| *Lythrum salicaria* | L. | Spike (of cymes) |
| *Malus domestica* | Borkh. | Corymb |
| *Malus sylvestris* | (L.) Mill. | Corymb |
| *Malva neglecta* | Wallr. | Raceme |
| *Malva sylvestris* | L. | Cyme |
| *Matricaria discoidea* | DC. | Solitary (capitulum) |
| *Medicago lupulina* | L. | Raceme |
| *Medicago sativa* | L. | Raceme |
| *Melilotus altissimus* | Thuill. | Raceme |
| *Mentha aquatica* | L. | Solitary |
| *Myosotis arvensis* | (L.) Hill | Cyme |
| *Onobrychis viciifolia* | Scop. | Raceme |
| *Origanum vulgare* | L. | Spike |
| *Papaver rhoeas* | L. | Solitary |
| *Pentaglottis sempervirens* | (L.) Tausch ex L.H.Bailey | Cyme |
| *Phacelia tanacetifolia* | Benth. | Cyme |
| *Potentilla anserina* | L. | Solitary |
| *Primula veris* | L. | Umbel |
| *Primula vulgaris* | Huds. | Solitary |
| *Prunella vulgaris* | L. | Spike |
| *Prunus avium* | (L.) L. | Umbel |
| *Prunus domestica* | L. | Cyme |
| *Prunus* sp. | NA | Cyme |
| *Prunus spinosa* | L. | Solitary |
| *Pulicaria dysenterica* | (L.) Bernh. | Corymb (of capitula) |
| *Pulicaria vulgaris* | Gaertn. | Corymb |
| *Pyrus communis* | L. | Corymb |
| *Ranunculus acris* | L. | Cyme |
| *Ranunculus repens* | L. | Cyme |
| *Rhinanthus minor* | L. | Spike |
| *Rosa acicularis* | Lindl. | Solitary |
| *Rosa canina* | L. | Corymb |
| *Rosa* sp. | NA | Solitary |
| *Rubus fruticosus* | L. | Raceme |
| *Salvia* sp. | NA | Spike |
| *Sambucus nigra* | L. | Cyme |
| *Sanicula europaea* | L. | Umbel |
| *Scorzoneroides autumnalis* | (L.) Moench. | Solitary (capitulum) |
| *Scrophularia nodosa* | L. | Panicle |
| *Senecio vulgaris* | L. | Cyme |
| *Silene dioica* | (L.) Clairv. | Cyme |
| *Silene latifolia* | Poir. | Cyme |
| *Silene vulgaris* | (Moench) Garcke | Cyme |
| *Sinapsis arvensis* | L. | Raceme |
| *Solanum dulcamara* | L. | Cyme |
| *Sonchus arvensis* | L. | Corymb |
| *Sonchus oleraceus* | NA | Umbel |
| *Sonchus* sp. | L. | Solitary |
| *Stachys sylvatica* | L. | Spike |
| *Stellaria graminea* | L. | Cyme |
| *Stellaria holostea* | L. | Cyme |
| *Stellaria media* | (L.) Vill. | Cyme |
| *Succisa pratensis* | Moench | Solitary (capitulum) |
| *Symphytum officinale* | L. | Cyme |
| *Syringa* sp. | NA | Panicle |
| *Syringa vulgaris* | L. | Panicle |
| *Tanacetum parthenium* | (L.) Sch. Bip. | Corymb |
| *Tanacetum vulgare* | L. | Corymb |
| *Taraxum officinale* | F. H. Wigg. | Solitary (capitulum) |
| *Teesdalia nudicaulis* | (L.) W. T. Aiton | Raceme |
| *Thlaspi arvense* | L. | Raceme |
| *Thymus vulgaris* | L. | Spike |
| *Tilia* sp. | NA | Cyme |
| *Torilis japonica* | (Houtt.) DC. | Umbel |
| *Trifolium incarnatum* | L. | Solitary (capitulum) |
| *Trifolium pratense* | L. | Solitary (capitulum) |
| *Trifolium repens* | L. | Solitary (capitulum) |
| *Trifolium resupinatum* | L. | Solitary (capitulum) |
| *Tripleurospermum inodorum* | (L.) Sch.Bip. | Solitary (capitulum) |
| *Ulex europaeus* | L. | Solitary |
| *Valeriana officinalis* | L. | Cyme |
| *Veronica arvensis* | L. | Raceme |
| *Veronica chamaedrys* | L. | Raceme |
| *Veronica persica* | Poir. | Solitary |
| *Viburnum* sp. | NA | Cyme |
| *Vicia cracca* | L. | Raceme |
| *Vicia faba* | L. | Solitary |
| *Vicia sativa* | L. | Solitary |
| *Vicia sepium* | L. | Raceme |
| *Vinca major* | L. | Solitary |
| *Viola arvensis* | Murray | Solitary |
| *Viola arvensis* | Murray | Solitary |
